# ﻿Revisiting *Szeptyckitheca* Betsch & Weiner (Collembola, Symphypleona, Sminthuridae): new species, updated diagnoses, and a key

**DOI:** 10.3897/zookeys.1186.111837

**Published:** 2023-12-12

**Authors:** Bruno Cavalcante Bellini, Mariana Fernandes De Oliveira, Wanda Maria Weiner, Rudy Camilo Nunes, Gleyce Da Silva Medeiros

**Affiliations:** 1 Department of Botany and Zoology, Biosciences Center, Federal University of Rio Grande do Norte (UFRN), Highway BR-101, Lagoa Nova, Campus Universitário, Natal 59072-970, RN, Brazil Federal University of Rio Grande do Norte Natal Brazil; 2 Institute of Systematics and Evolution of Animals, Polish Academy of Sciences, Sławkowska 17, Pl – 31 - 016 Kraków, Poland Institute of Systematics and Evolution of Animals, Polish Academy of Sciences Kraków Poland; 3 Biodiversity and Biotechnology Research Group of North Center Piaui, Federal Institute of Education, Science and Technology of Piaui, Pedro II 64255-000, Piaui, Brazil Federal Institute of Education, Science and Technology of Piaui Piaui Brazil

**Keywords:** Chaetotaxy, Neotropical fauna, *Sphyrotheca* Börner, Sphyrothecinae, taxonomy

## Abstract

*Szeptyckitheca* Betsch & Weiner is a genus of Sphyrothecinae (Sminthuridae) similar to *Sphyrotheca* Börner, with 13 nominal species. Most descriptions of *Szeptyckitheca* taxa lack valuable data in face of the current taxonomy of Symphypleona. In this study the previously described species of the genus were surveyed, aiming to provide updated diagnoses for them. Three species were also transferred to *Szeptyckitheca*: *Sphyrothecakarlarum* Palacios-Vargas, Vázquez & Cuéllar, 2003, *S.peteri* Palacios-Vargas, Vázquez & Cuéllar, 2003, and *S.koreana* Betsch & Weiner, 2009, based on trochanteral and/or dental chaetotaxy. Two new Brazilian species of the genus are described and illustrated, *S.andrzeji* Medeiros, Bellini & Weiner, **sp. nov.**, with a remarkable reduced ventral dental chaetotaxy not seen in other Neotropical species, and *S.cyanea* Oliveira, Medeiros & Bellini, **sp. nov.** with a distinctive large set of head vertex spines (18). Finally, an updated key to all the valid species of the genus is presented.

## ﻿Introduction

*Szeptyckitheca* Betsch & Weiner, 2009 is the second largest genus of the small subfamily Sphyrothecinae (Sminthuridae) with 13 described species, after *Sphyrotheca* Börner, 1906 with 21 (Bellinger et al. 1996–2023). The genus was erected to gather *S.kesongensis* Betsch & Weiner, 2009 and taxa previously assigned to *Sphyrotheca*, but with a trochanteral spine in the first pair of legs (usually in the trochanter II as well), anterior (ventral) dental chaetotaxy with three or four transversal lines of chaetae (one distal, one or two subdistal and one basal), cephalic chaeta **A1** often absent, and lacking clearly curved rough macrochaetae on frontal area of the head and dorsal large abdomen (Betsch 1980; Betsch and Weiner 2009). Further characteristics are presented ahead in the updated diagnosis of the genus.

*Szeptyckitheca* has a mainly Holotropical distribution, with few species occurring from other regions but under subtropical climates (Bellinger et al. 1996–2023; Betsch and Weiner 2009; Zeppelini et al. 2018). Such distribution is remarkably similar to that of *Sphyrotheca*; however, this last genus also holds a few taxa recorded from more temperate regions of the Holarctic (Bellinger et al. 1996–2023; Bretfeld 1999). As most Symphypleona lineages, the genus and its diagnostic features were never tested under a modern phylogenetic approach (Medeiros et al. 2022; Bellini et al. 2023), so the genus validity is solely based on its morphological description and its listed differences from other Sphyrothecinae, specially *Sphyrotheca*. Even so, as noted by Zeppelini et al. (2018), most species of *Szeptyckitheca* need to be redescribed or their diagnoses should be expanded, since many taxa were poorly described and many features used in the modern taxonomy of Symphypleona are not available for them.

Here we described in detail two new species of *Szeptyckitheca* from Brazil. We surveyed previously described taxa of the genus to present the current state of the knowledge of the group. We also transferred *Sphyrothecakarlarum* Palacios-Vargas, Vázquez & Cuéllar, 2003, *S.peteri* Palacios-Vargas, Vázquez & Cuéllar, 2003, and *S.koreana* Betsch & Weiner, 2009 to *Szeptyckitheca*. With this study we were able to present update diagnoses of *Szeptyckitheca* and its species, provide some taxonomic notes, and an updated identification key for them.

## ﻿Materials and methods

The studied specimens of *Szeptyckitheca* were preserved in 70% ethanol at 6 °C, clarified in Nesbitt’s solution, washed in Arlé’s liquid, and mounted on glass slides in Hoyer’s medium, combining procedures outlined by Arlé and Mendonça (1982) and Jordana et al. (1997). Habitus of the new species were photographed using a Leica EC4 camera attached to a Leica S8APO stereomicroscope, under LAS v. 4.12 software. Drawings were firstly made under a Leica DM750 microscope with a drawing tube, and photographs of the structures were taken with a Leica MC170 HD camera under LAS v. 4.12 software. Final figures were prepared with CorelDraw 2022 software.

For our survey of the previously described *Szeptyckitheca* species, we consulted the original descriptions as well as the redescription of *S.santiagoi* (Yosii, 1959) by Lawrence (1968). For the species distributions, we also checked Bellinger et al. (1996–2023), Mari-Mutt and Bellinger (1990, 1996), Mari-Mutt et al. (1997–2021), and Greenslade (2023).

The terminology used in the diagnoses and descriptions follows Cipola et al. (2014) for the labral chaetotaxy; Fjellberg (1999) for the labial palp; Betsch and Waller (1994) for the head and anterior large abdomen chaetotaxy; Vargovitsh (2009, 2012, 2013) for the posterior large abdomen chaetotaxy, with adaptations; and Betsch (1997) for the small abdomen chaetotaxy. Drawings and observations of the new species were made based on the entire type series.

Abbreviations used in the descriptions and figures are: **Abd.** —abdominal segment(s); **Ant.** —antennal segment(s); **mac** —macrochaeta(e); **Th.** —thoracic segment(s).

On the figures, chaetae present or absent are marked with white arrows; unpaired chaetae on head and trunk are marked with an asterisk, *. Ant. IV subsegments are counted from the base to the apex. The head vertex was considered as the frontal and interantennal areas sensu Betsch and Waller (1994) combined. Dens dorsal chaetae were considered as the dorsal, internal, and external chaetae rows combined. We considered as spines the strong and mostly stiff, pointed, blunt, or capitate/knobbed modified chaetae seen in the head vertex, trochanters, and femur I (the curved chaeta), following Bretfeld (1999) and Betsch and Weiner (2009).

Head, trunk (thorax + abdomen), and furca chaetotaxy are given by half body, with the exception of head vertex chaetae which are listed as their total number. Chaetae labels are marked in bold.

The type series of the new species are deposited at the Collembola Collection of the Biosciences Center of the Federal University of Rio Grande do Norte (**CC/UFRN**), Brazil.

## ﻿Results

### ﻿Taxonomic account

**Order Symphypleona Börner, 1901 sensu Bretfeld, 1986**

**Suborder Appendiciphora Bretfeld, 1986**

**Superfamily Sminthuroidea Bretfeld, 1994**

**Family Sminthuridae Lubbock, 1862**

**Subfamily SphyrothecinaeBetsch 1980**

#### ﻿ Szeptyckitheca

Taxon classificationAnimaliaSymphypleonaSminthuridae

Betsch & Weiner, 2009

5FCD4534-4D07-5107-AA90-CDE7A6319F16

##### Genus diagnosis.

Cuticle granulation rough. Specimens pigmented, eyepatches dark. Ant. IV with 8–12 subsegments. Eyes 8+8, head without vesicles or tubercles, eyepatches with 0–2 interocular chaetae, head vertex with a total of 4–18 strong erect large spines, cephalic chaeta **A1** present or absent. Large abdomen without mesothoracic vesicles, dorsally with spine-like, blunt, or curved mac, adults with bothriotricha **A**–**D**, parafurcal area with 1+1 neosminthuroid chaetae. Small abdomen without bothriotrichum **E**, female subanal appendage short (not reaching the apex of the ventral anal valves), long (surpassing the ventral anal valves) or very long (surpassing the dorsal anal valve). Trochanteral spines present on legs I and III, sometimes on leg II as well, ungues usually with the internal tooth, pseudonychia and tunica. Manubrium with six or seven dorsal chaetae. Ventral (anterior) dental chaetotaxy with two to four transversal rows of chaetae, with the following resumed chaetotaxy formula from the apex to the base: 4–2,2–0,2–0…1, dorsal dens with 13–24 chaetae. Mucro without chaeta, with an apical notch (data based on Denis 1948; Yosii 1959, 1965, 1966; Delamare-Deboutteville and Massoud 1964a, b; Betsch 1965; Hüther 1967; Lawrence 1968; Snider 1978; Bretfeld 2005; Betsch and Weiner 2009; Zeppelini et al. 2018).

##### Type species.

*Szeptyckithecakesongensis* Betsch & Weiner, 2009.

##### Remarks.

The closest genus to *Szeptyckitheca* is *Sphyrotheca*, and many features in their diagnoses overlap (Betsch and Weiner 2009). They share a subsegmented Ant. IV, Th. II without vesicles, the presence of bothriotrichum **D** in adult specimens, and the trochanteral spine in leg III (Bretfeld 1999; Betsch and Weiner 2009). Here we considered as the main unequivocal diagnostic feature to separate both genera the presence of at least one trochanteral spine in leg I of *Szeptyckitheca* (absent in *Sphyrotheca*) (Betsch and Weiner 2009). Other features like the reduction of the ventral dens chaetotaxy and presence of **A1** cephalic chaeta vary within *Szeptyckitheca*, as well as the presence of curved mac on dorsal head and large abdomen of *Sphyrotheca*, and cannot be used alone to separate the genera (Tables 1, 2; see also the Discussion).

**Table 1.** Main features of *Szeptyckitheca* species from the Americas.

Species / features	*S.andrzeji* sp. nov.	*S.cyanea* sp. nov.	*S.bellingeri*	*S.kac*	*S.karlarum*	*S.mucroserrata*	*S.peteri*	*S.vanderdrifti*
Trunk color pattern	pinkish, with purple dorsal spots	dark bluish	dorsally dark brownish with lateral sides bluish	with dark spots	♂ pale bluish with purple spots and stripes, ♀ yellowish with brown spots and stripes	with lateral weak purple bands, posteriorly purple	♂ pale bluish with purple spots, ♀ yellowish with brown spots	yellowish with indistinct areas of pigment, or brownish
Ant. IV subsegments	11	11	8	9	9–10	9–10	9–10	~10
Ant. III chaetae	19	21**	18	14	19	?	17?	19
Ant. II chaetae	13	15	14?	13	15	?	12	14
Ant. II long chaetae	3	4	3	3	2	?	2	2–3
Ant. I chaetae	6	6	5?	7	6	?	4?	6
Interocular chaetae	-	2	2	-	2	2	1	2
Frontal head **A1**	-	+	-	-	-	-	-	-
Head vertex large spines (total)	14	18	14	16	16	16	16	16
Head vertex rough spines	-	-	-?	-?	+	+	+	-
Head frons sec. reduced chaetae	+	-	+	+	+	-?	+	+
Head interantennal bifid chaetae (♀)	-	-	-	-	-	-	-	-
Head clypeal **f** line mac	-	-	-	4	-	-	-	-
Tr. I n° of spines	2	2	2	1	1	?	1	1
Tr. I spine(s) apex shape	capitate	capitate	acuminate	capitate	blunt	?	?	acuminate
Tr. II spine apex shape	blunt	capitate	blunt	knobbed	-	?	knobbed	acuminate?
Tr. III spine apex shape	blunt	blunt	blunt	blunt	blunt	blunt	blunt	capitate
Tr. III regular chaetae	5	5	5	3	5	5	5	4
Tr. III oval organs	1	1	-?	-	1	+	1	-?
Femur I spine	+(1)	+(1)	+(1)	+(1)	+(1)	?	+(1)	?
Ungues inner tooth	+	+	+	+	+	+	+	+
Ungual tunica	+	+	+	+	+	+	+	+
Ungual pseudonychia	weak	weak	strong	weak	+	-	+	strong
Unguiculus I internal tooth	-	+	-	+	+	-	+	+
Unguiculus III apical filament	thin	thin	thick	thin	thin	thin	thin	thin
Unguiculus III filament length compared to unguis III	=	<	>	=	<	=	>	<
Large abdomen capitate mac	+	+	-	+	+	-	+*	-
Female anal valve **mps1** winged	-	-	-	-	-	-	-	-
Female subanal appendage shape	short, spoon-like, apically serrated	long, slightly curved at the apex, unilaterally serrated	long, slightly curved at the apex, smooth or unilaterally serrated	long, blunt, apically serrated	long, spatulated, serrated at the tip	long, spatulated or acuminated, serrated at the tip	long, spatulated, serrated at the tip	long, acuminate, apically or almost entirely serrated
Manubrial chaetae	7	7	6	?	?	7	?	?
Dens ventral chaetotaxy	3…1	3,2…1	2,2…1	4,1...1	3,2…1	3,2…1^§^	3,2…1	2,2…1
Dens dorsal chaetae	17	16	17	15	12	14^§^	17	16
Prominent mucronal notch	-	+	+	+	-	-	-	-

Data based on the original descriptions. Legends: ‘sec.’ secondarily, ‘Tr.’ trochanter; ‘+’ present; ‘-’ absent; ‘=’ subequal; ‘>’ surpassing the tip of unguis; ‘<’ not reaching the tip of unguis; ‘?’ unknown, unclear data; ‘*’ described as mesochaetae; ‘**’ = including the peculiar small sensilla on cavities outside the apical organ; ‘^§’ =^ the proximal chaeta portrayed in the ventral dens (Snider 1978: 235, fig. 207) is lateral and large, and does not correspond to the typical reduced proximal ventral chaeta seen in other species of the genus. Due to position and size, we believe this chaeta was represented in the dorsal dens (Snider 1978: 235, fig. 208).

**Table 2.** Main features of *Szeptyckitheca* species from the Old World.

Species / features	*S.boneti*	*S.coerulea*	*S.formosana*	*S.implicata*	*S.kesongensis*	*S.koreana*	*S.machadoi*	*S.nepalica*	*S.santiagoi*	*S.spinimucronata*
Trunk color pattern	dorsally black or purplish-red	bluish with white spots	dorsally yellowish with lateral sides purplish	dark yellowish with purple stripes and spots	pale	lateral large abdomen violet	with transversal stripes and spots of dark pigment	pale, with diffused pigment and violet patches	variable, usually laterally dark and dorsally pale	whitish, with diffused fields of purple pigment
Ant. IV subsegments	10–12	9–10	9–10	11	10	9	10	10	10	~10
Ant. III chaetae	24*	?	?	?	23	23	21*	?	15?	?
Ant. II chaetae	?	15–16	?	>8	16	16	15*	?	15?	?
Ant. II long chaetae	?	-?	?	-	-	-	4	-?	1	?
Ant. I chaetae	?	7	4?	5	7	7	5?	?	5	?
Interocular chaetae	2	2	-?	1	2	2	2?	-?	1	-?
Frontal head **A1**	+	+	+	+	-	+	+	+	-	+
Head vertex large spines (total)	10	6	15	16	16	4****	16	11	14 or 16	16
Head vertex rough spines	-?	-	+	+	+/-	+/-	-?	-?	+?	+
Head frons sec. reduced chaetae	+	-	-?	-	-	+	-	-?	+?	-
Head interantennal bifid chaetae (♀)	-	-	-	-	+	-	-	-	-	-
Head clypeal **f** line mac	-	-	-	-	-	-	-	-	-	-
Tr. I n° of spines	?	1	?	1	1	1	1	1	1	?
Tr. I spine apex shape	?	blunt	?	blunt	capitate	capitate	blunt	blunt	blunt	?
Tr. II spine apex shape	?	blunt	?	blunt	capitate	capitate	-	blunt	blunt	?
Tr. III spine apex shape	?	blunt	?	blunt	capitate	capitate	blunt	blunt	blunt	blunt
Tr. III regular chaetae	?	5	?	5	5	4	4	?	5	5
Tr. III oval organs	?	-?	?	-?	2	2	-	?	?	-?
Femur I spine	?	+(1)	?	?	+(1)	+(1)	+(1)	?	+(2)	?
Ungues inner tooth	+	+	+	+	+	+	+	+	-	+
Ungual tunica	-**	+	+	+	+	+	+	+	+	+
Ungual pseudonychia	weak	strong	-	weak	strong	strong	strong	strong	weak	-
Unguiculus I internal tooth	+	-	+	+	-	+	+	+/-	+/-	-
Unguiculus III apical filament	thin	thin	thin	thin	thin	thin	thin	thin?	thin	thin
Unguiculus III filament compared to unguis III	<	<	<	<	<	=	>	<	=	<
Large abdomen capitate mac	?	+	-	+	-	-	?	-	-	-
Female anal valve **mps1** winged	-	-	-	-	+	-	-	-	-	-
Female subanal appendage shape	very long, acuminate, serrated at the middle	long, acuminate, unilaterally serrated	long, blunt, apically serrated	long, acuminate, apically serrated	long, acuminate, apically serrated	long, blunt, apically unilaterally serrated	long, spatulated, apically serrated	long, acuminate, apically serrated	long, with a bidentate apex, smooth or apically serrated	long, acuminate, apically serrated
Manubrial chaetae	?	7	7	7	?	?	?	6	?	7
Dens ventral chaetotaxy	3,2,2…1 or 3,2…1	4,2…1	3,1…1	2,2…1	3,2…1	3…1 or 2…1	2,2…1	3,2....1	2,2…1***	3,2…1
Dens dorsal chaetae	17–18	16	15	19	20	17	24	13	13***	17
Prominent mucronal notch	+	+	+	+	+	+	+	+	+	+

Data based on the original descriptions, with exception of *S.santiagoi*, which was based on Yosii (1959) and Lawrence (1968). Legends: ‘sec.’ secondarily, ‘Tr.’ trochanter; ‘+’ present; ‘-’ absent; ‘~’ about; ‘=’ subequal; ‘>’ surpassing the tip of unguis; ‘<’ not reaching the tip of unguis; ‘?’ unknown, unclear data; ‘*’ = including the peculiar small sensilla on cavities outside the apical organ; ‘**’ = ungues I and II without tunica, unguis III possibly with a small distal rudiment; ‘***’ = we considered dental chaetae as described by Yosii (1959), since Lawrence’s (1968) redescription is imprecise regarding this feature and may be mistaken; ‘****’ = the large head vertex spines of *S.koreana*, comb. nov. are mixed with large curved rough mac.

In our survey and the descriptions of the new species we observed some species hold reduced spines on the frontal head. We considered such reduction as secondary, since other taxa of the genus present all frontal head chaetae well developed, a feature also seen in other genera and families of Symphypleona (Betsch 1980; Bretfeld 1999).

###### ﻿Species diagnoses

#### ﻿ Szeptyckitheca bellingeri

Taxon classificationAnimaliaSymphypleonaSminthuridae

(Betsch, 1965)

DF2C163D-4D01-574D-8B01-C1A1B5819C94

Sphyrotheca bellingeri Betsch, 1965: 444.

##### Diagnosis.

Yellowish ground, head with dark blue bands dorsally in a V-shape, dorsal trunk dark brownish with bronze, bluish or yellowish effects, other regions including appendages bluish. Ant. IV with 8 subsegments; Ant. III with 18 chaetae other than the sensory clubs; Ant. II with three chaetae clearly longer than the others. Eyepatches with two interocular chaetae modified into strong spines each. Head vertex with a total of 14 large spines, two of them unpaired; unpaired chaeta **A1** absent; secondarily reduced chaeta near the spines present. Trochanters I–III with 2,1,1 spines, respectively, trochanter I spines acuminate, trochanters II and III spines blunt; trochanter III with five regular chaetae other than the spine. Ungues with a single inner tooth, with tunica and strong pseudonychia; unguiculus I without the internal tooth; unguiculus III filament thick and surpassing the tip of the unguis III. Large abdomen lacking capitate mac. Female with a long subanal appendage (surpassing the ventral anal valves), slightly curved at the apex (hook-like), smooth or with the external border serrated. Manubrium with 6+6 dorsal chaetae; dens ventral chaetotaxy formula from the apex to the base as: 2,2…1, dorsal chaetotaxy with 17 chaetae; mucronal notch prominent (adapted from Betsch 1965).

##### Remarks.

*Szeptyckithecabellingeri* is the sole species of the genus with eight subsegments of the Ant. IV, while all others have nine or more. It also shares with the two new described species a pair of trochanteral spines on leg 1, a feature not reported in any other taxon of the genus (see Tables 1, 2).

##### Habitat.

Specimens were found on mosses and liverworts growing on logs and stony ground, over the vegetation, litter layer, dead branches and directly upon the soil (Betsch 1965).

##### Known distribution.

Jamaica (Betsch 1965).

#### ﻿ Szeptyckitheca boneti

Taxon classificationAnimaliaSymphypleonaSminthuridae

(Denis, 1948)

79355B19-BB4B-5971-9ACC-97F04889F5B8

*Sminthurus Boneti* [sic] Denis, 1948: 298.

##### Diagnosis.

Dorso-posterior head and dorsal trunk blackish to purplish-red, legs and antennae weakly pigmented, ventral side pale. Ant. IV with 10–12 subsegments; Ant. III with 24 chaetae other than the sensory clubs, including two peculiar small sensilla within cavities. Eyepatches with two regular interocular chaetae each. Head vertex with a total of ten large spines, two of them unpaired; unpaired chaeta **A1** present; secondarily reduced chaetae near the spines present. Ungues with a single inner tooth, without tunica (unguis III possibly with a small distal rudiment of tunica) and with a weakly developed pseudonychia; unguiculus I with the internal tooth; unguiculus III filament thin and not reaching the tip of the unguis III. Female with a very long subanal appendage (surpassing the dorsal anal valve), acuminate, feathered (serrated) at the middle region on its both edges. Dens ventral chaetotaxy formula from the apex to the base as: 3,2…1 or 3,2,2…1, dorsal chaetotaxy with 17 or 18 chaetae; mucronal notch prominent (adapted from Denis 1948).

##### Remarks.

*Szeptyckithecaboneti* is unique compared to all its congeners by the very long subanal appendages of the females, surpassing the dorsal anal valves. However, we could not find any data on the species legs chaetotaxy, especially the presence and shape of the trochanteral spines. Apparently the species was revised by Betsch (Betsch 1980; Betsch and Weiner 2009) before its inclusion in the genus, but it is in need of redescription, since many important data on its morphology are unknown (see Table 2).

##### Habitat.

Specimens were found in bushes (Denis 1948).

##### Known distribution.

Vietnam (Denis 1948).

#### ﻿ Szeptyckitheca coerulea

Taxon classificationAnimaliaSymphypleonaSminthuridae

(Bretfeld 2005)

7031443F-EADA-523B-A0C9-6D71438B8B0F

Sphyrotheca coerulea Bretfeld, 2005: 32.

##### Diagnosis.

Head and body almost completely blue, with pale spots. Ant. IV with nine or ten subsegments; Ant. II with 15 or 16 chaetae; Ant. I with seven chaetae. Eyepatches with two regular interocular chaetae each. Head vertex with a total of six large smooth spines, none of them unpaired; unpaired chaeta **A1** present; secondarily reduced chaetae near the spines absent. Trochanters I–III with 1,1,1 spines, respectively, all blunt; trochanter III with five regular chaetae other than the spine. Ungues with a single inner tooth, with tunica and strong pseudonychia; unguiculus I without the internal tooth; unguiculus III filament thin and not reaching the tip of the unguis III. Large abdomen dorsally with several capitate mac. Female with a long subanal appendage (surpassing the ventral anal valves), acuminate, apically serrated on its internal face. Manubrium with 7+7 dorsal chaetae; dens ventral chaetotaxy formula from the apex to the base as: 4,2…1, dorsal chaetotaxy with 16 chaetae; mucronal notch prominent (adapted from Bretfeld 2005).

##### Remarks.

*Szeptyckithecacoerulea* is the only species of the genus with six spines on the head vertex. Further data on the species are presented in Table 2.

##### Habitat.

Specimens were found on shrubs and grasses near a stream of water (Bretfeld 2005).

##### Known distribution.

Yemen (Socotra Island) (Bretfeld 2005).

#### ﻿ Szeptyckitheca formosana

Taxon classificationAnimaliaSymphypleonaSminthuridae

(Yosii, 1965)

83ED4651-27EF-58D0-B545-8BD0E72DB11D

Sphyrotheca formosana Yosii, 1965: 49.

##### Diagnosis.

Yellowish ground, antennae diffusely pigmented, head almost pale, large abdomen laterally purplish, dorsally pale. Ant. IV with nine or ten subsegments. Head vertex with a total of 15 large spines, three of them unpaired; unpaired chaeta **A1** present. Ungues with a single inner tooth, with tunica and without pseudonychia; unguiculus I with the internal tooth; unguiculus III filament thin and not reaching the tip of the unguis III. Large abdomen without capitate chaetae. Female’s subanal appendage long (surpassing the ventral anal valves), blunt, apically serrated on its external face. Dens ventral chaetotaxy formula from the apex to the base as: 3,1…1, dorsal chaetotaxy with 15 chaetae; mucronal notch prominent (adapted from Yosii 1965).

##### Remarks.

Like *S.boneti*, *S.formosana* was apparently revised by Betsch (Betsch 1980; Betsch and Weiner 2009). Nevertheless, many important data on its morphology are still lacking (see Table 2), and it is in need of redescription.

##### Habitat.

Unknown.

##### Known distribution.

Taiwan (Yosii 1965).

#### ﻿ Szeptyckitheca implicata

Taxon classificationAnimaliaSymphypleonaSminthuridae

(Hüther, 1967)

3FF0ED45-6B99-5B56-8099-0F2F80BBD53E

Sphyrotheca implicata Hüther, 1967: 252.

##### Diagnosis.

Dark yellowish ground, with dark purple spots and two longitudinal stripes on the dorsal large abdomen, appendages pale purple. Ant. IV with 11 subsegments; Ant. II with more than eight chaetae, none of them clearly longer than the others; Ant. I with five chaetae. Eyepatches with one regular interocular chaeta each. Head vertex with a total of 16 large spines, two of them unpaired; unpaired chaeta **A1** present; secondarily reduced chaetae near the spines absent. Trochanters I–III with 1,1,1 spines, respectively, all blunt; trochanter III with five regular chaetae other than the spine. Ungues with a single inner tooth, with tunica and weak pseudonychia; unguiculus I with the internal tooth; unguiculus III filament thin and not reaching the tip of the unguis III. Large abdomen dorsally with several capitate mac. Female with a long subanal appendage (surpassing the ventral anal valves), acuminate, apically serrated. Manubrium with 7+7 chaetae, dens ventral chaetotaxy formula from the apex to the base as: 2,2…1, dorsal chaetotaxy with 19 chaetae; mucronal notch prominent (adapted from Hüther 1967).

##### Habitat.

Specimens were found associated to bushes (Hüther 1967).

##### Known distribution.

South Sudan (Hüther 1967).

#### ﻿ Szeptyckitheca kac

Taxon classificationAnimaliaSymphypleonaSminthuridae

Zeppelini, Lopes & Lima, 2018

4ADF2244-9130-52D8-86C0-579DA546420B

Szeptyckitheca kac Zeppelini, Lopes & Lima, 2018: 3.

##### Diagnosis.

Trunk with dark spots of pigment, mucro and legs less pigmented. Ant. IV with nine subsegments; Ant. III with 14 chaetae other than the sensory clubs; Ant. II with 13 chaetae, three of them clearly longer than the others; Ant. I with seven chaetae; some chaetae of Ant. II–IV capitate. Eyepatches lacking interocular chaetae. Head vertex with a total of 16 large spines, two of them unpaired; unpaired chaeta **A1** absent; secondarily reduced chaetae near the spines present. Clypeal area with 4+4 mac near the antennae. Trochanters I–III with 1,1,1 spines, respectively, trochanter I spine capitate, II knobbed and III blunt; trochanter III with three regular chaetae other than the spine. Ungues with a single inner tooth, with tunica and weak pseudonychia; unguiculus I with the internal tooth; unguiculus III filament thin and reaching the tip of the unguis III. Large abdomen dorsally with several capitate mac. Female with a long subanal appendage (surpassing the ventral anal valves), blunt, apically serrated on its internal face. Dens ventral chaetotaxy formula from the apex to the base as: 4,1…1, dorsal chaetotaxy with 15 chaetae; mucronal notch prominent (adapted from Zeppelini et al. 2018).

##### Remarks.

*Szeptyckithecakac* is unique within the genus due the presence of 4+4 modified mac on the upper clypeus (**f** line) and the dens ventral chaetotaxy formula of 4,1...1 chaetae. Further data on the species are presented in Table I.

##### Habitat.

Specimens were found in the canopy of rainforest (Zeppelini et al. 2018).

##### Known distribution.

Brazil (Zeppelini et al. 2018).

#### ﻿ Szeptyckitheca karlarum

Taxon classificationAnimaliaSymphypleonaSminthuridae

(Palacios-Vargas, Vázquez & Cuéllar, 2003) comb. nov.

DAF87AE6-B382-546F-BA80-62CCD4AE2217

Sphyrotheca karlarum Palacios-Vargas, Vázquez & Cuéllar, 2003: 303–306, 308, figs 4–6, Mexico, Quintana Roo, Reserva de la Biosfera de Sian Ka’na (orig. descr.).

##### Diagnosis.

Males pale bluish, with purple or blue spots on antennae, dorsal head and furca, dorsal trunk striped; females yellowish, with brown spots and stripes with the same distribution of males. Ant. IV with nine or ten subsegments, with some proximal chaetae capitate; Ant. III with 19 chaetae other than the sensory clubs, 4–6 of them longer than the others and capitate; Ant. II with 15 chaetae, two of them clearly longer than the others, two of them modified into spines; Ant I with six chaetae. Eyepatches with two interocular chaetae each, one of them modified into a spine. Head vertex with a total of 16 large and rough spines, two of them unpaired; unpaired chaeta **A1** absent; secondarily reduced chaetae near the spines present. Trochanters I–III with 1,0,1 spines, respectively, trochanters I and III spines blunt; trochanter III with five regular chaetae other than the spine. Ungues with a single inner tooth, with tunica and strong pseudonychia; unguiculus I with the internal tooth; unguiculus III filament thin and not reaching the tip of the unguis III. Large abdomen with capitate chaetae. Female with a long subanal appendage (slightly surpassing the ventral anal valves), spatulated, apically serrated on both faces. Dens ventral chaetotaxy formula from the apex to the base as: 3,2…1, dorsal chaetotaxy with 12 chaetae; mucronal notch discrete (adapted from Palacios-Vargas et al. 2003).

##### Remarks.

*Sphyrothecakarlarum* is herein transferred to *Szeptyckitheca* due to the presence of robust and mostly erect spines on the head vertex and dorsal large abdomen, presence of three transversal rows of dental ventral chaetae and presence of spines on trochanters I and III, features used by Betsch and Weiner (2009) to separate *Szeptyckitheca* from *Sphyrotheca*.

##### Habitat.

Specimens were found in low flooded jungle (Palacios-Vargas et al. 2003).

##### Known distribution.

Mexico (Palacios-Vargas et al. 2003).

#### ﻿ Szeptyckitheca kesongensis

Taxon classificationAnimaliaSymphypleonaSminthuridae

Betsch & Weiner, 2009

CE8739D0-88AC-526E-962D-B20C4A333FC4

Szeptyckitheca kesongensis Betsch & Weiner, 2009: 40.

##### Diagnosis.

Specimens pale or very clear. Ant. IV with ten subsegments; Ant. III with 23 chaetae other than the sensory clubs; Ant. II with 16 chaetae, none of them clearly longer than the others; Ant. I with seven chaetae. Eyepatches with two regular interocular chaeta each. Head vertex with a total of 16 large spines, two of them unpaired; unpaired chaeta **A1** absent; secondarily reduced chaetae near the spines absent. Head interantennal area with 1+1 bifid chaetae. Trochanters I–III with 1,1,1 spines, respectively, all capitate; trochanter III with five regular chaetae other than the spine. Ungues with a single inner tooth, with tunica and strong pseudonychia; unguiculus I without the internal tooth; unguiculus III filament thin and not reaching the tip of the unguis III. Large abdomen without capitate mac. Female with a long subanal appendage (surpassing the ventral anal valves), acuminate, apically serrated on both edges. Dens ventral chaetotaxy formula from the apex to the base as: 3,2…1, dorsal chaetotaxy with 20 chaetae; mucronal notch prominent (adapted from Betsch and Weiner 2009).

##### Remarks.

*Szeptyckithecakesongensis*, the type species of the genus, shows a very peculiar head morphology, with the female bearing bifid chaetae in the interantennal area, while the male lack such morphology (Betsch and Weiner 2009). Although only one couple of specimens was analyzed for this description, no other *Szeptyckitheca* species has such a sexual dimorphism, including the species herein described.

##### Habitat.

Specimens were found associated to *Robinia* sp., shrubs, pines, and plant debris (Betsch and Weiner 2009).

##### Known distribution.

North Korea (Betsch and Weiner 2009).

#### ﻿ Szeptyckitheca koreana

Taxon classificationAnimaliaSymphypleonaSminthuridae

(Betsch & Weiner, 2009) comb. nov.

80631E6B-B624-5BB9-B30F-F501B748A505

Sphyrotheca koreana Betsch & Weiner, 2009: 36–39, figs 1–13, North Korea, Kaesong-si province, Chonma-sun Mountains (orig. descr.).

##### Diagnosis.

Pale background, with antennae, frontal area (interocular field) and lateral sides of large abdomen violet. Ant. IV with nine subsegments; Ant. III with 23 chaetae other than the sensory clubs; Ant. II with 16 chaetae, none of them clearly longer than the others; Ant. I with seven chaetae. Eyepatches with two regular interocular chaetae each. Head vertex with a total of four large erect spines, plus four short spines and nine rough curved mac, three of them unpaired; unpaired chaeta **A1** present; secondarily reduced chaetae near the spines present. Trochanters I–III with 1,1,1 spines, respectively, all capitate; trochanter III with four regular chaetae other than the spine. Ungues with a single inner tooth, with tunica and strong pseudonychia; unguiculus I with the internal tooth; unguiculus III filament thin and reaching the tip of the unguis III. Large abdomen without capitate mac, but with rough curved mac. Female with a long subanal appendage (surpassing the ventral anal valves), blunt, apically serrated on its internal edge. Dens ventral chaetotaxy formula from the apex to the base as: 3…1 or 2…1, dorsal chaetotaxy with 17 chaetae; mucronal notch prominent (adapted from Betsch and Weiner 2009).

##### Remarks.

*Sphyrothecakoreana* is herein transferred to *Szeptyckitheca* due to the presence of one spine on trochanters I–III. This feature, which was listed as diagnostic of the later genus by Betsch and Weiner (2009), was overlooked in the original paper possibly due to the overall morphology of the species, which, disregarding the trochanteral chaetotaxy, better matches *Sphyrotheca*. This includes the presence of large rough curved chaetae on the head vertex and dorsal large abdomen and only two transversal lines of ventral dens chaetae (Betsch and Weiner 2009). However, since we observed some species of *Szeptyckitheca* with reduced ventral dens chaetotaxy, and taxa listed as *Sphyrotheca* species without the dorsal rough curved mac, we provisionally transfer *Sphyrothecakoreana* to *Szeptyckitheca*. Nevertheless, this intermediate species placement should be better investigated in the light of modern phylogenetic methods to better understand its position within the Sphyrothecinae. Further data on the boundaries of *Szeptyckitheca* and *Sphyrotheca* are discussed ahead in the text.

##### Habitat.

Specimens were found in forested areas (Betsch and Weiner 2009).

##### Known distribution.

North Korea (Betsch and Weiner 2009).

#### ﻿ Szeptyckitheca machadoi

Taxon classificationAnimaliaSymphypleonaSminthuridae

(Delamare-Deboutteville & Massoud, 1964)

8B967407-4577-515B-8C9C-5491D17ACFE7

Sminthurotheca machadoi Delamare-Deboutteville & Massoud, 1964a: 80.

##### Diagnosis.

Specimens with transversal stripes and spots of dark pigment. Ant. IV with ten subsegments; Ant. III with 21 chaetae other than the sensory clubs, two of them as small sensilla in individual cavities; Ant. II with 15 chaetae, one of them as a small sensillum in cavity, four of the regular chaetae clearly longer than the others. Head vertex with a total of 16 large spines, two of them unpaired; unpaired chaeta **A1** present; secondarily reduced chaetae near the spines absent. Trochanters I–III with 1,0,1 spines, respectively, trochanters I and III spines blunt; trochanter III with four regular chaetae other than the spine. Ungues with a single inner tooth, with tunica and strong pseudonychia; unguiculus I with the internal tooth; unguiculus III filament thin and surpassing the tip of the unguis III. Female with a long subanal appendage (surpassing the ventral anal valves), spatulated, apically serrated on both edges. Dens ventral chaetotaxy formula from the apex to the base as: 2,2…1, dorsal chaetotaxy with 24 chaetae; mucronal notch prominent (adapted from Delamare-Deboutteville and Massoud 1964a).

##### Remarks.

The genus *Sminthurotheca* Delamare-Deboutteville & Massoud, 1964 was erected based on a supposedly unique combination of Ant. III and large abdomen chaetotaxy. It was posteriorly synonymized with *Sphyrotheca* by Betsch (1980), due to the overlapping morphology of both genera. Later, *Sphyrothecamachadoi* was tranfered to *Szeptyckitheca* by Betsch and Weiner (2009), especially due to the presence of a spine on the trochanter I and the ventral dens chaetotaxy with three whorls of chaetae. Further data on the species are presented in Table 2.

##### Habitat.

Specimens were found in gallery forests, in plant debris (Delamare-Deboutteville and Massoud 1964a).

##### Known distribution.

Angola, Congo (Delamare-Deboutteville and Massoud 1964a).

#### ﻿ Szeptyckitheca mucroserrata

Taxon classificationAnimaliaSymphypleonaSminthuridae

(Snider, 1978)

E0B3CA88-DEBA-5991-98C8-3E45A881594C

Sphyrotheca mucroserratus Snider, 1978: 236.

##### Diagnosis.

Antennae, legs, and furca purplish, head purplish near the mouth, with purple bands between the antennae and on its vertex, trunk laterally with pale purple bands, posterior abdomen purplish. Ant. IV with nine or ten subsegments. Eyepatches with two interocular chaetae modified into strong spines each. Head vertex with a total of 16 large spines, two of them unpaired; unpaired chaeta **A1** absent. Trochanter III spine blunt, with five extra regular chaetae. Ungues with a single inner tooth, with tunica but lacking pseudonychia; unguiculus I without the internal tooth; unguiculus III filament thin and reaching the tip of the unguis III. Large abdomen lacking capitate mac. Female with a long subanal appendage (surpassing the ventral anal valves), spatulated or acuminated, serrated at the tip. Manubrium with 7+7 dorsal chaetae; dens ventral chaetotaxy formula from the apex to the base as: 3,2…1, dorsal chaetotaxy with 14 chaetae; mucronal notch discrete (adapted from Snider 1978).

##### Remarks.

The short description of Snider (1978) of *Sphyrothecamucroserratus* limits the comparison of this species with its congeners. It lacks many important useful taxonomical features (see Table 1), including some recognized as diagnostic of the genus, like the presence of the trochanter I spine and even the pair of neosminthuroid chaetae which are diagnostic of the Sphyrothecinae. The wide distribution of the species, recorded from all Americas, combined with its generic and imprecise diagnosis, support the hypothesis that the name *Szeptyckithecamucroserrata* has possibly been used to circumscribe a complex of species. In this scenario its redescription is urgent, to improve the comprehension of the morphology and distribution of *Szeptyckitheca* species from the New World.

##### Habitat.

Specimens listed in the original description were found associated with Australian pine needles, leaf mold, and forest debris in Florida, USA (Snider 1978). In Brazil, specimens of *S.mucroserrata* were found associated to forest litter and on sand dunes (Abrantes et al. 2012).

##### Known distribution.

Brazil, Mexico, and USA (Snider 1978; Mari-Mutt and Bellinger 1996; Abrantes et al. 2012).

#### ﻿ Szeptyckitheca nepalica

Taxon classificationAnimaliaSymphypleonaSminthuridae

(Yosii, 1966)

512F3FEA-CFAC-5B28-B5AD-4BBE73FC8679

Sphyrotheca nepalica Yosii, 1966: 527.

##### Diagnosis.

Pale ground, body diffusely pigmented with brownish violet patches between the eyes and lateral sides of the large abdomen, antennae distally dark pigmented. Ant. IV with ten subsegments. Head vertex with a total of 11 large spines, three of them unpaired, including chaeta **A1**. Trochanters I–III with 1,1,1 spines, respectively, all blunt. Ungues with a single inner tooth, with tunica and strong pseudonychia; unguiculus I with or without the internal tooth; unguiculus III filament not reaching the tip of the unguis III. Large abdomen without capitate mac. Female with a long subanal appendage (surpassing the ventral anal valves), acuminate, apically serrated on both edges. Manubrium with 6+6 dorsal chaetae; dens ventral chaetotaxy formula from the apex to the base as: 3,2…1, dorsal chaetotaxy with 13 chaetae; mucronal notch prominent (adapted from Yosii 1966).

##### Remarks.

*Szeptyckithecanepalica* is the only species of the genus with 11 spines on head vertex. Although the species fits *Szeptyckitheca*, especially due to the presence of the spines on trochanters I–III, its description is quite limited considering the current taxonomy of Symphypleona (see Table 2), and the species needs a formal redescription, as already noted by Betsch and Weiner (2009).

##### Habitat.

Unknown.

##### Known distribution.

Nepal (Yosii 1966).

#### ﻿ Szeptyckitheca peteri

Taxon classificationAnimaliaSymphypleonaSminthuridae

(Palacios-Vargas, Vázquez & Cuéllar, 2003) comb. nov.

582EBBB3-A161-501D-B9F1-CBD0BAFBB127

Sphyrotheca peteri Palacios-Vargas, Vázquez & Cuéllar, 2003: 298–302, figs 1–3, Mexico, Quintana Roo, Reserva de la Biosfera de Sian Ka’na (orig. descr.).

##### Diagnosis.

Males pale bluish, with purple or blue spots on antennae, dorsal head, dorsal trunk and furca, females yellowish, with brown spots with the same distribution of males. Ant. IV with nine or ten subsegments, with some proximal chaetae capitate; Ant. II with 12 chaetae, two of them clearly longer than the others. Eyepatches with one interocular chaeta each. Head vertex with a total of 16 large and rough spines, two of them unpaired; unpaired chaeta **A1** absent; secondarily reduced chaetae near the spines present. Trochanters I–III with 1,1,1 spines respectively, trochanter II spine knobbed and III blunt; trochanter III with five regular chaetae other than the spine. Ungues with a single inner tooth, with tunica and strong pseudonychia; unguiculus I with the internal tooth; unguiculus III filament thin and surpassing the tip of the unguis III. Large abdomen with capitate chaetae. Female with a long subanal appendage (surpassing the ventral anal valves), spatulated, apically serrated on both faces. Dens ventral chaetotaxy formula from the apex to the base as: 3,2…1, dorsal chaetotaxy with 17 chaetae; mucronal notch discrete (adapted from Palacios-Vargas et al. 2003).

##### Remarks.

*Sphyrothecapeteri* is herein transferred to *Szeptyckitheca* due to the presence of robust and somewhat erect spines on the head vertex and dorsal large abdomen, presence of three transversal rows of dental ventral chaetae and presence of spines on all trochanters, all features originally listed by Betsch and Weiner (2009) as diagnostic of *Szeptyckitheca*.

##### Habitat.

Specimens were found in low flooded jungle (Palacios-Vargas et al. 2003).

##### Known distribution.

Mexico (Palacios-Vargas et al. 2003).

#### ﻿ Szeptyckitheca santiagoi

Taxon classificationAnimaliaSymphypleonaSminthuridae

(Yosii, 1959)

99663B62-A173-5E6B-AF95-B11B72584C2A

Sphyrotheca santiagoi Yosii, 1959: 58; Lawrence 1968: 333.

##### Diagnosis.

Color pattern variable, usually mostly dark with a pale dorsum. Ant. IV with ten subsegments; Ant. III with at least 15 chaetae other than the sensory clubs; Ant. II with at least 15 chaetae, one of them clearly longer than the others; Ant. I with five chaetae. Eyepatches with one interocular somewhat spine-like chaeta each. Head vertex with a total of 14 or 16 spines, two of them unpaired; unpaired chaeta **A1** absent. Trochanters I–III with 1,1,1 spines, respectively, all blunt; trochanter III with five regular chaetae other than the spine. Ungues without the inner tooth, with tunica and weak pseudonychia; unguiculus I with or without the internal tooth; unguiculus III filament thin and reaching the tip of the unguis III. Large abdomen without capitate mac. Female with a long subanal appendage, bidentate at the apex, smooth or apically serrated. Dens ventral chaetotaxy formula from the apex to the base as: 2,2…1, dorsal chaetotaxy with 13 chaetae; mucronal notch prominent (adapted from Yosii 1959 and Lawrence 1968).

##### Remarks.

*Szeptyckithecasantiagoi* is the sole species of the genus without the ungual inner tooth. However, the variability of color patterns reported by Lawrence (1968), discrepancies in its redescription compared to the original one of Yosii (1959), like differences in unguiculus morphology and dental chaetotaxy, and the wide distribution of the species in different islands of Asia and Oceania (Lawrence 1968) suggest the name *S.santiagoi* hides a species complex. In this sense, the diagnosis herein provided and the data listed in Table 2 for this species should be taken as provisional until the species can be redescribed.

##### Habitat.

Specimens were found in forest moss and litter, beach debris, up palms and in native gardens (Lawrence 1968).

##### Known distribution.

Australia, Papua New Guinea, Singapore, Solomon Islands (Yosii 1959; Lawrence 1968; Greenslade 2023).

#### ﻿? Szeptyckitheca spinimucronata

Taxon classificationAnimaliaSymphypleonaSminthuridae

(Itoh, 1993)

52A3A105-A6A1-59C9-8FC8-B5C171FE297A

Sphyrotheca spinimucronata Itoh, 1993, in Itoh and Zhao 1993: 33.

##### Diagnosis.

White ground with diffuse purple pigment on anterior and dorsal head and dorsal and lateral trunk, antennae darker. Ant. IV with ~ 10 subsegments. Head vertex with a total of 16 large spines, two of them unpaired; unpaired chaeta **A1** present; secondarily reduced chaetae near the spines absent. Trochanter III spine blunt, with five extra regular chaetae. Ungues with a single inner tooth, with tunica and lacking pseudonychia; unguiculus I without the internal tooth; unguiculus III filament thin and not reaching the tip of the unguis III. Large abdomen without capitate mac. Female with a long subanal appendage (surpassing the ventral anal valves), acuminate, apically serrated on both edges. Manubrium with 7+7 dorsal chaetae; dens ventral chaetotaxy formula from the apex to the base as: 3,2…1, dorsal chaetotaxy with 17 chaetae; mucronal notch prominent (adapted from Itoh and Zhao 1993).

##### Remarks.

*Sphyrothecaspinimucronata* is listed as a *Szeptyckitheca* species in Bellinger et al. (1996–2023), possibly due to its resemblance with *S.nepalica* (Itoh and Zhao 1993), and that is why we give its diagnosis here. However, it was not cited in the original description of the genus or in the most recent key to the group (Betsch and Weiner 2009; Zeppelini et al. 2018). Without the confirmation of the presence of trochanters I and II spines and many other relevant morphological traits (see Table 2), the positioning of *S.spinimucronata* within *Szeptyckitheca* is doubtful, and we considered it as a *species inquirenda*.

##### Habitat.

Specimens were found in a coniferous forest of *Cryptomeriafortunei* (Itoh and Zhao 1993).

##### Known distribution.

China (Itoh and Zhao 1993).

#### ﻿ Szeptyckitheca vanderdrifti

Taxon classificationAnimaliaSymphypleonaSminthuridae

(Delamare-Deboutteville & Massoud, 1964)

A43C8798-3697-54A2-8057-911EAA3D08A7

Sphyrotheca vanderdrifti Delamare-Deboutteville & Massoud, 1964b: 64.

##### Diagnosis.

Yellowish ground color, with indistinct pigmented fields and purplish spots on the dorsum, antennae purplish. Ant. IV with ~ 10 subsegments; Ant. III with 19 chaetae other than the sensory clubs; Ant. II with 14 chaetae, two or three of them clearly longer than the others; Ant. I with six chaetae. Eyepatches with two interocular chaetae modified into strong spines each. Head vertex with a total of 16 large spines, two of them unpaired; unpaired chaeta **A1** absent; secondarily reduced chaeta near the spines present. Trochanters I–III with 1,1,1 spines, respectively, trochanter I spine acuminate and III capitate; trochanter III with four regular chaetae other than the spine. Ungues with a single inner tooth, with tunica and strong pseudonychia; unguiculus I with the internal tooth; unguiculus III filament thin and not reaching the tip of the unguis III. Large abdomen lacking capitate mac. Female with a long subanal appendage (surpassing the ventral anal valves), acuminate, and apically or almost entirely serrated on its both edges. Dens ventral chaetotaxy formula from the apex to the base as: 2,1…1, dorsal chaetotaxy with 16 chaetae; mucronal notch discrete (adapted from Delamare-Deboutteville and Massoud 1964b).

##### Habitat.

Specimens were found on marshy wood on sandy loam and shrubs on a ridge (Delamare-Deboutteville and Massoud 1964b).

##### Known distribution.

Suriname (Delamare-Deboutteville and Massoud 1964b).

#### ﻿ Szeptyckitheca andrzeji

Taxon classificationAnimaliaSymphypleonaSminthuridae

Medeiros, Bellini & Weiner sp. nov.

757DC0B6-2D0F-5A1C-B6B1-B932CA82D324

https://zoobank.org/35417D56-6F55-4B77-A90F-576C05A36816

**Figure 1.**
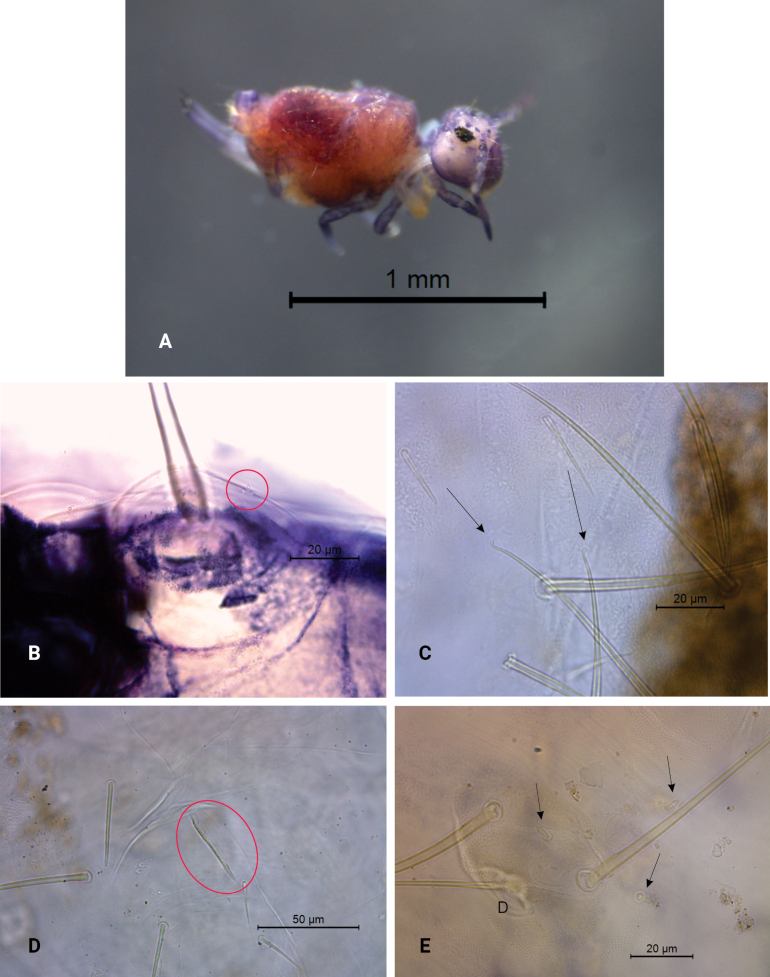
*Szeptyckithecaandrzeji* sp. nov. **A** habitus in ethanol (dorsal view) **B** frontal head spine on papilla, red circle marks a secondarily reduced spine **C** large abdomen capitate mac**D** parafurcal area, red circle marks the neosminthuroid chaeta **E** bothriotrichum D, black arrows indicate small blunt accessory chaetae.

**Figure 2.**
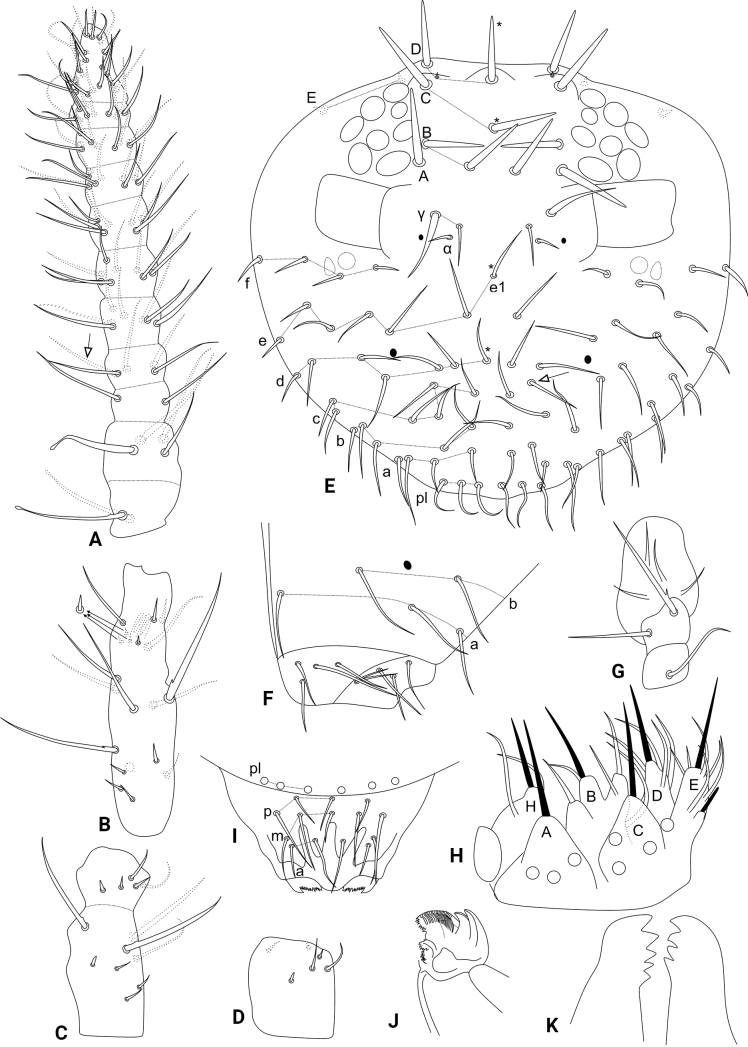
*Szeptyckithecaandrzeji* sp. nov. head **A** dorsal Ant. IV (white arrow points to chaeta present or absent) **B** dorsal Ant. III **C** dorsal Ant. II **D** dorsal Ant. I **E** anterior head chaetotaxy and eyes **F** labial and postlabial (ventral) chaetotaxy (right side) **G** maxillary outer lobe and sublobal plate (right side) **H** labial papillae and proximal chaetae alveoli (right side) **I** labrum **J** maxilla capitulum (right side) **K** mandibles apexes.

**Figure 3.**
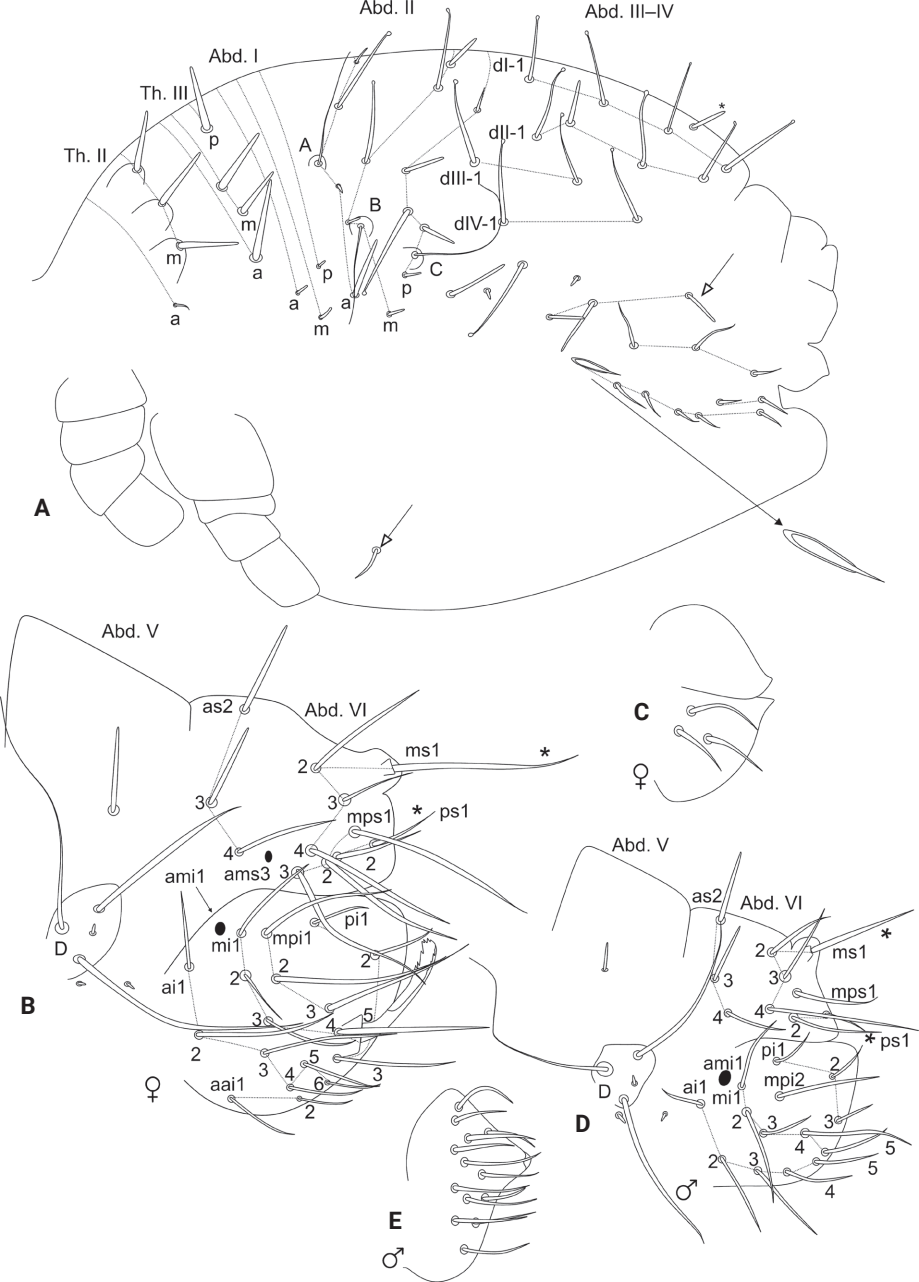
*Szeptyckithecaandrzeji* sp. nov. trunk chaetotaxy **A** male large abdomen, detail shows the neosminthuroid chaeta and its alveolus, chaeta marked with a white arrow can be missing **B** female small abdomen **C** female genital plate **D** male small abdomen **E** male genital plate.

**Figure 4.**
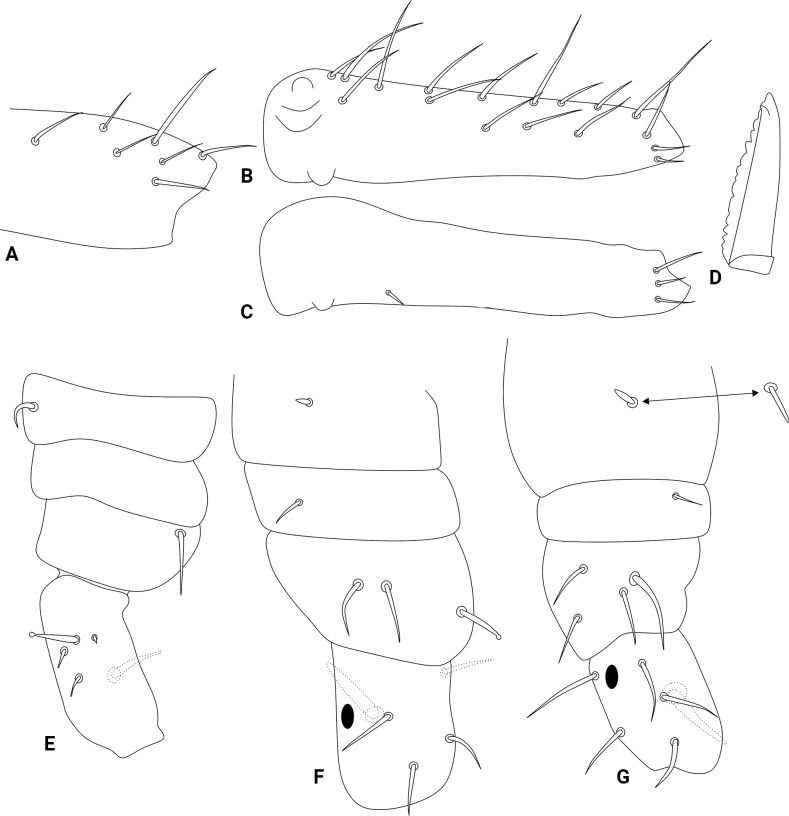
*Szeptyckithecaandrzeji* sp. nov. trunk appendages **A** manubrium **B** dorsal dens **C** ventral dens **D** mucro **E** epicoxa, subcoxa, and coxa of leg I **F** epicoxa, subcoxa, and coxa of leg II **G** epicoxa, subcoxa, and coxa of leg III, double arrow points to alternative morphology of the epicoxal chaeta.

**Figure 5.**
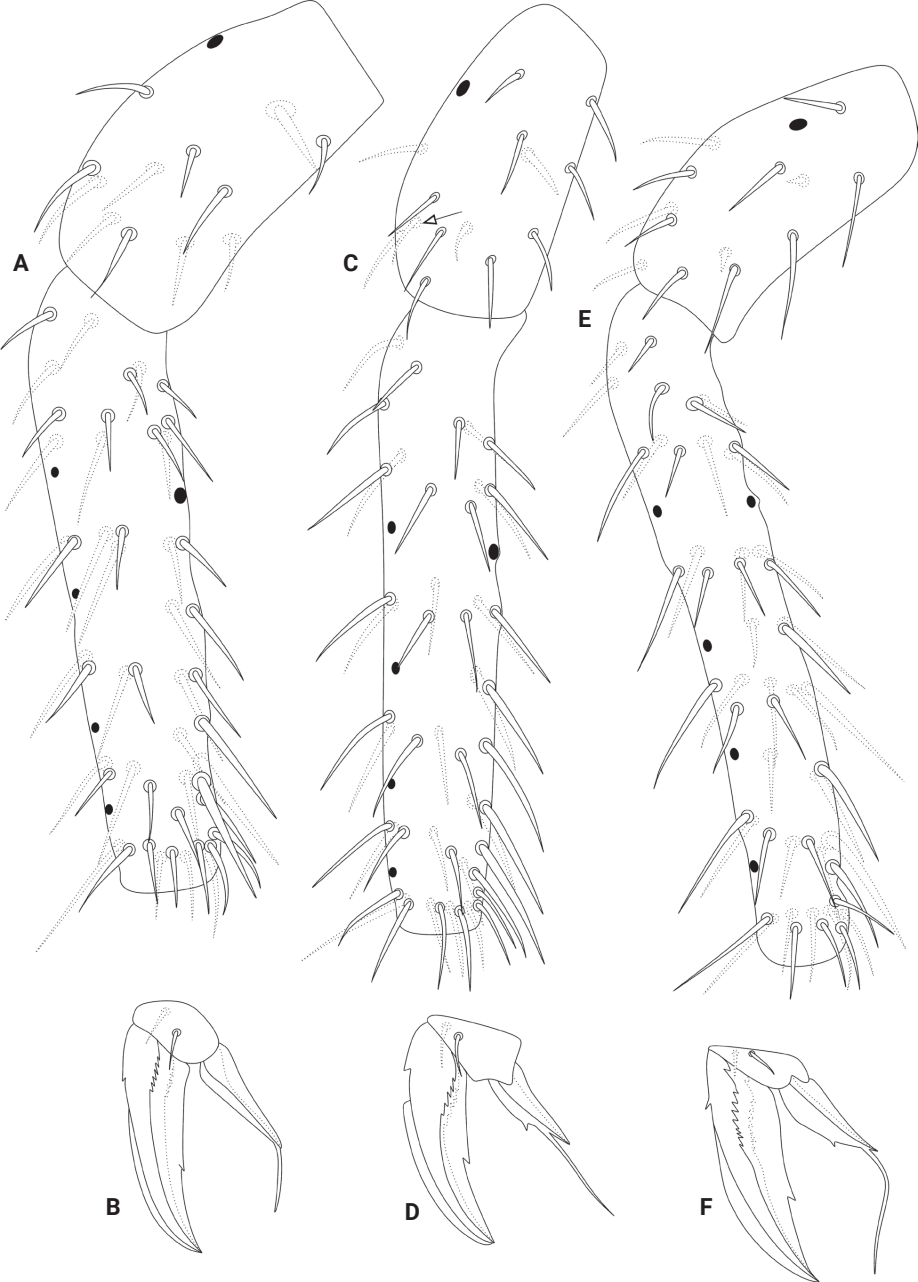
*Szeptyckithecaandrzeji* sp. nov. legs **A** femur and tibiotarsus I **B** foot complex I **C** femur and tibiotarsus II, chaeta marked with a white arrow can be missing **D** foot complex II **E** femur and tibiotarsus III **F** foot complex III.

**Figure 6.**
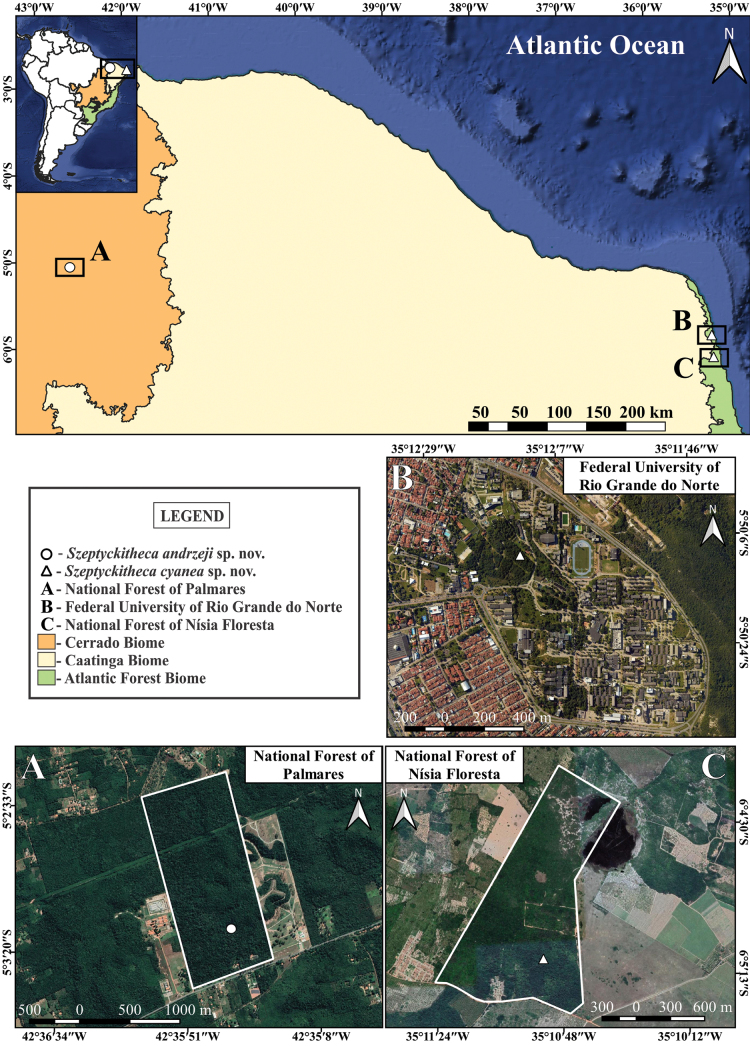
Known distribution of *Szeptyckithecaandrzeji* sp. nov. and *Szeptyckithecacyanea* sp. nov. in Brazil **A** National Forest of Palmares, in Altos municipality, Piauí state **B** Federal University of Rio Grande do Norte, in Natal city, Rio Grande do Norte state **C** National Forest of Nísia Floresta, in Nísia Floresta municipality, Rio Grande do Norte state. In **A** and **C**, the white line polygons delimit the sampled areas boundaries.

Figs 1 , 2 , 3 , 4 , 5 , 6 , Table 1

##### Type material.

***Holotype*** male on slide, Brazil, Piauí state, Altos municipality, “Floresta Nacional de Palmares” (5°3'12.53"S, 42°35'36.95"W), in sandy soil, Cerrado biome, 06/IV/2022, Mesquita C.P. col., pitfall traps. ***Paratypes*** on slides: one male, two females, one juvenile, with the same data as the holotype.

##### Diagnosis.

Ground color pinkish, with purple spots on head, dorso-anterior large abdomen, and dorsal small abdomen. Ant. IV with 11 subsegments, with five capitate chaetae; Ant. III with 19 chaetae other than the sensory clubs, two of them clearly longer than the others; Ant. II subdivided, with 13 chaetae, three of them clearly longer than the others; Ant. I with six chaetae. Eyepatches lacking interocular chaetae. Head vertex with a total of 14 large spines, two of them unpaired; unpaired chaeta **A1** absent; secondarily reduced chaetae near the spines present. Trochanters I–III with 2,1,1 spines, respectively, trochanter I spines capitate, II and III spines blunt; trochanter III with five regular chaetae other than the spine. Ungues with one internal tooth, with tunica and weak pseudonychia; unguiculus I without the internal tooth; unguiculus III filament thin and reaching the tip of the unguis III. Large abdomen dorsally with 15+15 long capitate mac. Female with a short subanal appendage (not reaching the apex of the ventral anal valves), spoon-like, and apically serrated on both faces. Manubrium with 7+7 dorsal chaetae; dens ventral chaetotaxy formula from the apex to the base as: 3…1, dorsal chaetotaxy with 17 chaetae; mucronal notch discrete.

##### Description.

Body (head + trunk) length of the type series ranging between 900 µm and 1400 µm, holotype with 900 µm, male average size = 900 µm, female average size = 1300 µm, entire type series average size = 1100 µm. Ground color pinkish, with purple spots on head, Ant. I–III, dorso-anterior large abdomen and dorsal small abdomen. Ant. IV, legs, and dens uniformly purplish (Fig. 1A).

Head (Figs 1B, 2). Antennae length 433 µm in the holotype. Holotype antennal segment ratio I:II:III:IV as 1:1.7:2.1:4.2. Ant. IV with 11 subsegments, subsegment I with two, II with four, III with two, IV with four or five, V with seven, VI with nine, VII–X with ten each, and XI with ~ 24 chaetae, respectively; subsegment I with two, subsegment II with three capitate chaetae respectively, some of them basally barbed (Fig. 2A). Ant. III with 19 chaetae other than the sensory rods, two of them clearly longer than the others and basally barbed, 11 reduced to some extent, one baso-ventral oval organ, sensory rods inside distinct shallow cavities (Fig. 2B). Ant. II subdivided, basal subsegment with eight, apical with five chaetae, respectively, basal subsegment with three enlarged chaetae, two of them basally barbed, Ant. II with seven chaetae reduced to some extent (Fig. 2C). Ant. I with six small chaetae, two of them ventral (Fig. 2D). Eyes 8+8, interocular chaetae absent, head capsule normal (not elongated) (Fig. 2E). Clypeal area **a**–**f** lines with 7/7/5–6/6(+1)/6(+1)/4 dorsal + ventral chaetae, respectively, **e1** chaeta present, 2+2 zones without cuticular granulation next to **f** line (Fig. 2E). Interantennal area **α** and **γ** lines with 1/2 chaetae, respectively, 1+1 spine-like, plus 1+1 oval organs; frontal area **A**–**E** lines with a total of 12 large smooth spines, chaetotaxy following the formula: 1/2/1(+1)/2(+1)/2, respectively, **D** and **E** lines with secondarily reduced spines, **A1** chaeta absent (Figs 1B, 2E). Labial basomedian field with four, basolateral field with five chaetae, respectively; cephalic groove with 1+1 surrounding chaetae from **a** line (Fig. 2F). Maxillary outer lobe with apical chaeta basally barbed, longer than the basal chaeta, sublobal plate with four chaeta-like appendages (Fig. 2G). Labial palp with seven proximal chaetae, formula of the guards: **H**(2), **A**(0), **B**(5), **C**(0), **D**(4), **E**(6) plus the lateral process (Fig. 2H). Six prelabral chaetae present (Fig. 2E); labral **p**, **m**, and **a** lines with 5, 5, 4 chaetae, respectively, **p2** longer than the others, labral intrusions present, labral papillae absent, labrum apically toothed (Fig. 2I). Mandibles normal (not elongated), with 5+4 incisive apical teeth (Fig. 2K). Maxilla capitulum spherical, without any clear modification (Fig. 2J).

Trunk (Figs 1C–E, 3). Large abdomen: male blunt chaetae on dorsal large abdomen and all chaetae of the parafurcal area shorter than in females. Thorax continuous with the abdomen, without segmentations. Th. II with one **a** small chaeta and three blunt spines on papillae on **m** line; Th. III with one **a**, two **m** and one **p** blunt spines; Abd. I with one **a**, one **m**, and one **p** short blunt chaetae; Abd. II bothriotricha **A**, **B**, and **C** slightly misaligned, all short, with four **a**, five **m**, and five **p** chaetae of different shapes near the bothriotricha, Abd. II with four long capitate mac. Abd. III–IV with one unpaired dorsal chaeta plus four main lines of chaetae above the bothriotrichum **C**: **dI-1** with four, **dII-1** with four, **dIII-1** with two, and **dIV-1** with two chaetae, respectively, 11 of them as four long capitate mac (Figs 1C, 3A). Parafurcal area with four rows of chaetae, with three, three, two and seven chaetae, respectively, neosminthuroid chaeta present (Figs 1D, 3A). Small abdomen: including Abd. V–VI in both sexes. Abd. V chaetae smooth, with bothriotrichum **D** surrounded by three small blunt plus two long chaetae, a blunt chaeta above bothriotrichum **D** elongate in females, short in males (Figs 1E, 3B, D), Abd. VI chaetae discretely serrated (not represented in the drawings). Female Abd. VI: dorsal anal valve with **as2**–**4**, **ms1**–**4**, **mps1**–**3**, and **ps1**–**2** chaetae, **ms1** and **ps1** unpaired, **ams3** as an oval organ; each ventral anal valve with **aai1**–**2**, **ai1**–**6**, **ami1** (as an oval organ), **mi1**–**5**, **mpi1**–**3**, and **pi1**–**3** chaetae, **mi5** as the subanal appendage, short (not reaching the apex of the ventral anal valves), spoon-like, and apically serrated on both faces (Fig. 3B). Female genital plate with 3+3 ventral chaetae (Fig. 3C). Male Abd. VI: dorsal anal valve with **as2**–**4**, **ms1**–**4**, **mps1**, and **ps1**–**2** chaetae, **ms1** and **ps1** unpaired; each ventral anal valve with **ai1**–**5**, **ami1** (as an oval organ), **mi1**–**5**, **mpi2**, and **pi1**–**3** chaetae (Fig. 3D). Male genital plate with 13+13 chaetae (Fig. 3E).

Abdominal appendages (Fig. 4A–D). Ventral tube with 1+1 chaetae on the lateral flaps, sacs long and warty. Tenaculum ramus with three teeth each plus an apically rounded basal appendix, corpus with 2+2 chaetae. Manubrium with 7+7 dorsal chaetae (Fig. 4A); dens dorsally with two basal rounded appendages and 17 dorsal (posterior) chaetae (Fig. 4B); dens ventrally (anteriorly) with four chaetae, following the formula from the apex to the basis: 3...1 (Fig. 4C). Mucro short, apically split, external lamella serrated (with 10–17 serrations), internal smooth, ending in a discrete tooth-like apical notch (Fig. 4D). Manubrium:dens:mucro ratio of the holotype = 1.1:2.5:1.

Legs (Figs 4E–G, 5). Leg I: epicoxa and coxa with one chaeta each, subcoxa without chaeta; trochanter with two capitate spines plus two capitate and one reduced acuminate chaetae (Fig. 4E); femur with one oval organ, one acuminate spine and 11 regular chaetae; tibiotarsus with five oval organs and 49 chaetae, ten of them in the apical whorl (Fig. 5A); pretarsus with one anterior and one posterior chaetae with similar sizes, unguis with one internal and one dorsal teeth, lateral teeth absent, with tunica and weak pseudonychia, unguiculus without tooth, apical filament thin and not reaching the tip of the unguis (Fig. 5B). Leg II: epicoxa and subcoxa with one chaeta each, coxa with three chaetae, one of them capitate; trochanter with one thick blunt spine plus one oval organ and four regular chaetae (Fig. 4F); femur with one oval organ, one acuminate spine and 12 or 13 regular chaetae; tibiotarsus with five oval organs and 47 chaetae, 10 of them in the apical whorl (Fig. 5C); pretarsus with one anterior and one posterior chaetae with similar sizes, unguis with one internal and one dorsal teeth, lateral teeth absent, with tunica and weak pseudonychia, unguiculus with an internal tooth, apical filament thin and reaching the tip of the unguis (Fig. 5D). Leg III: epicoxa and subcoxa with one chaeta each, coxa with four chaetae; trochanter with one thick blunt spine, one oval organ and five regular chaetae (Fig. 4G); femur with one oval organ, two reduced and 10 regular chaetae; tibiotarsus with five oval organs and 50 chaetae, ten of them in the apical whorl (Fig. 5E); pretarsus with one anterior and one posterior chaetae with similar sizes, unguis with one internal and one dorsal teeth, lateral teeth absent, with tunica and weak pseudonychia, unguiculus with one internal tooth, apical filament thin and reaching the tip of the unguis (Fig. 5F); tibiotarsi oval organs without reduced inner chaetae. Ratio of ungues I–III in the holotype = 1:1.08:1.19.

##### Etymology.

The species honors Dr. Andrzej Szeptycki for his important contributions to the taxonomy and systematics of springtails.

##### Habitat.

Specimens of *Szeptyckithecaandrzeji* sp. nov. were collected in the National Forest of Palmares, a small federal conservation unit, with a total area of 168.21 hectares, and altitudes ranging between 154 m and 250 m, located in Altos municipality, Piauí state, close to Teresina, the state’s capital (Fig. 6). The conservation unit is inserted in the Parnaíba River Sedimentary Basin, within the limits of the Cerrado biome, with influence of the Caatinga e Amazon biomes (ICMBio 2022). The vegetation of the region consists of a seasonal semideciduous forest, locally known as “Forest Cerrado” or “Cerradão” (Miranda et al. 2005), with a tree layer of medium to large size, reaching ~ 15–20 meters in height.

The climate in the region, according to the Köppen–Geiger climate classification system, is Aw, megathermic tropical with a long dry season and rainy summer, with high temperatures throughout the year (Kottek et al. 2006). Between 1991 and 2020, the average annual temperature was 28–30 °C, with the minimum between 22–24 °C and the maximum between 33–35 °C, with an average annual insolation of 2800–3000 hours. The average annual precipitation was 1400–1600 mm, with February, March, and April as the rainiest quarter and July, August, and September as the driest one. The annual potential evapotranspiration was 2400–2600 mm, with a marked water deficit, which is typical of this Brazilian region (INMET 2023). The soil in the site where the specimens were collected is of the Latosol type, deep and well evolved, with some gravel on the surface, coming from nearby slopes, where young, shallow, and rocky soils predominate (ICMBio 2022).

##### Remarks.

*Szeptyckithecaandrzeji* sp. nov. is unique among the Neotropical taxa due to its reduced ventral dens chaetotaxy, with only four chaetae distributed in two transversal rows, following the formula from the apex to the basis: 3...1. All other Neotropical *Szeptyckitheca* species have three transversal lines of dental ventral chaetae. Also, this is the only species of the genus with a short subanal appendage, not reaching the apex of the ventral anal valves (Tables 1, 2). The new species is somewhat similar to *S.kac* Zeppelini, Lopes & Lima, 2018 in color pattern, Ant. III chaetotaxy, and absence of interocular chaetae, but differs in the above-mentioned features, Ant. I chaetae (6 in the new species, 7 in *S.kac*), absence of lateral mac on **f** line on *S.andrzeji* sp. nov. (vs presence), shape of the trochanteral spines and number of regular chaetae on trochanter III (5 vs 3) and on dorsal dens (17 vs 15). Further comparisons are presented in Tables 1 and 2.

#### ﻿ Szeptyckitheca cyanea

Taxon classificationAnimaliaSymphypleonaSminthuridae

Oliveira, Medeiros & Bellini sp. nov.

5C078AB1-E856-532E-B5F2-09F69958BDB8

https://zoobank.org/7A5516D9-4A67-40CA-B470-4EEAFA975D47

**Figure 7.**
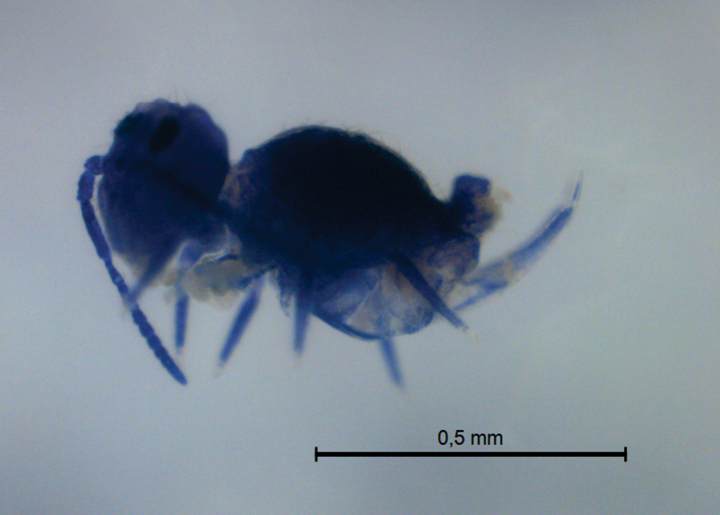
*Szeptyckithecacyanea* sp. nov. habitus in ethanol (lateral view).

**Figure 8.**
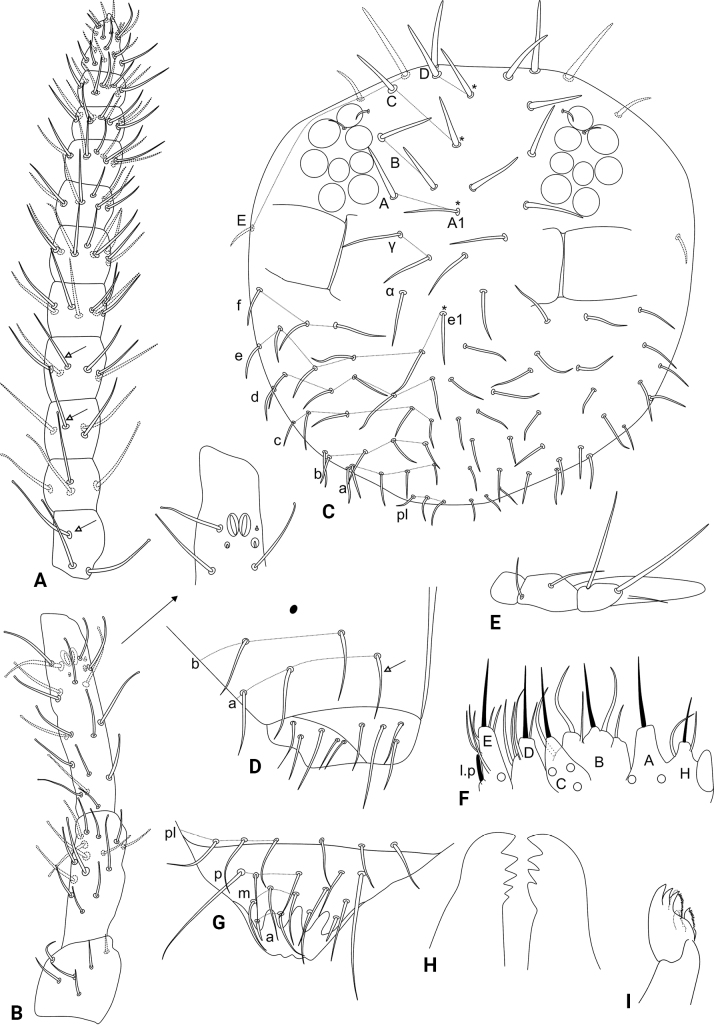
*Szeptyckithecacyanea* sp. nov. head **A** dorsal Ant. IV (white arrows point to chaetae present or absent) **B** dorsal Ant. I–III, detail shows the ventral apical organ and the small sensilla in cavities on Ant. III **C** anterior head chaetotaxy and eyes **D** labial and postlabial (ventral) chaetotaxy (left side), white arrow points to chaeta present or absent **E** maxillary outer lobe and sublobal plate (left side) **F** labial papillae and proximal chaetae alveoli (left side) **G** labrum **H** mandibles apexes **I** maxilla capitulum (left side).

**Figure 9.**
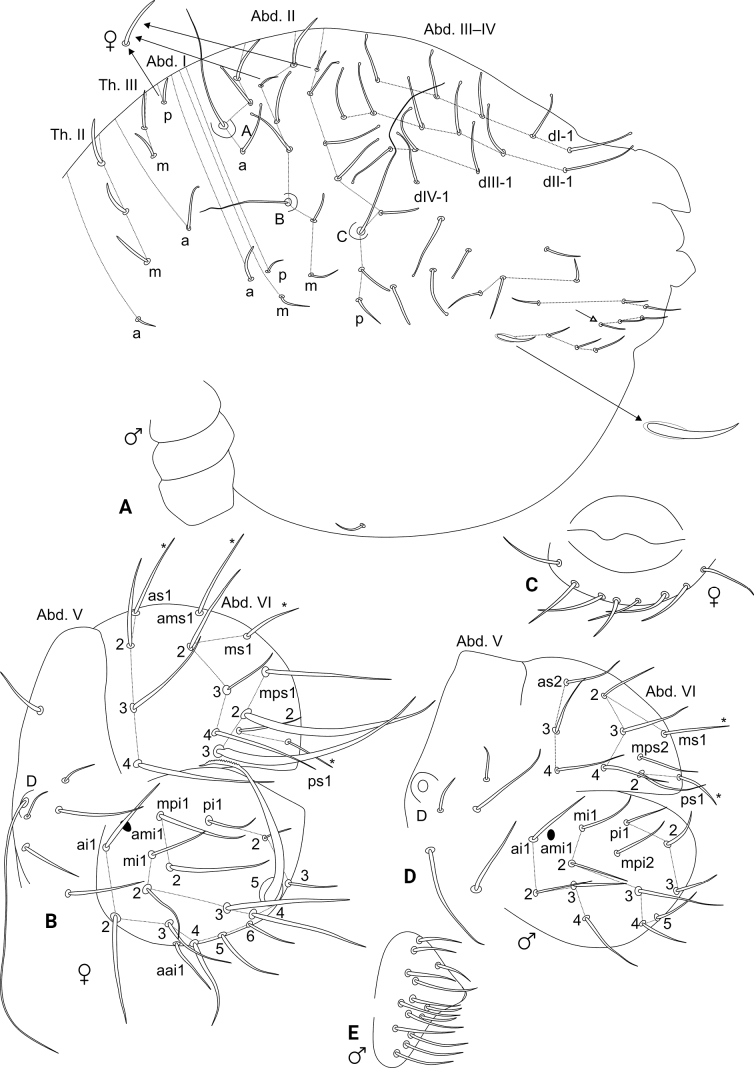
*Szeptyckithecacyanea* sp. nov. trunk chaetotaxy **A** male large abdomen, details show the neosminthuroid chaeta and its alveolus and longer chaetae of the female **B** female small abdomen **C** female genital plate **D** male small abdomen **E** male genital plate.

**Figure 10.**
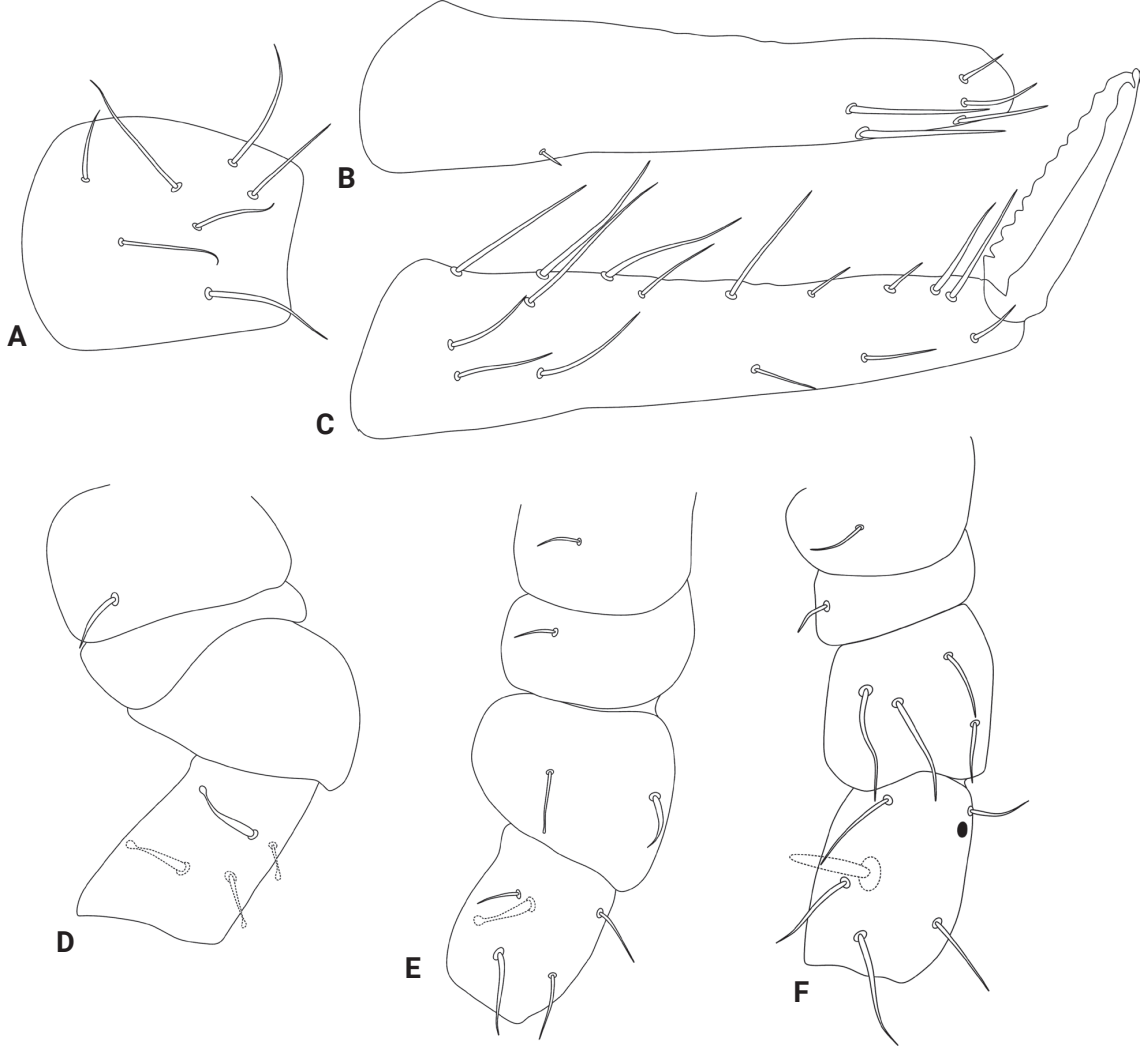
*Szeptyckithecacyanea* sp. nov. trunk appendages **A** manubrium **B** ventral dens **C** dorsal dens and mucro **D** epicoxa, subcoxa, and coxa of leg I **E** epicoxa, subcoxa, and coxa of leg II **F** epicoxa, subcoxa, and coxa of leg III.

**Figure 11.**
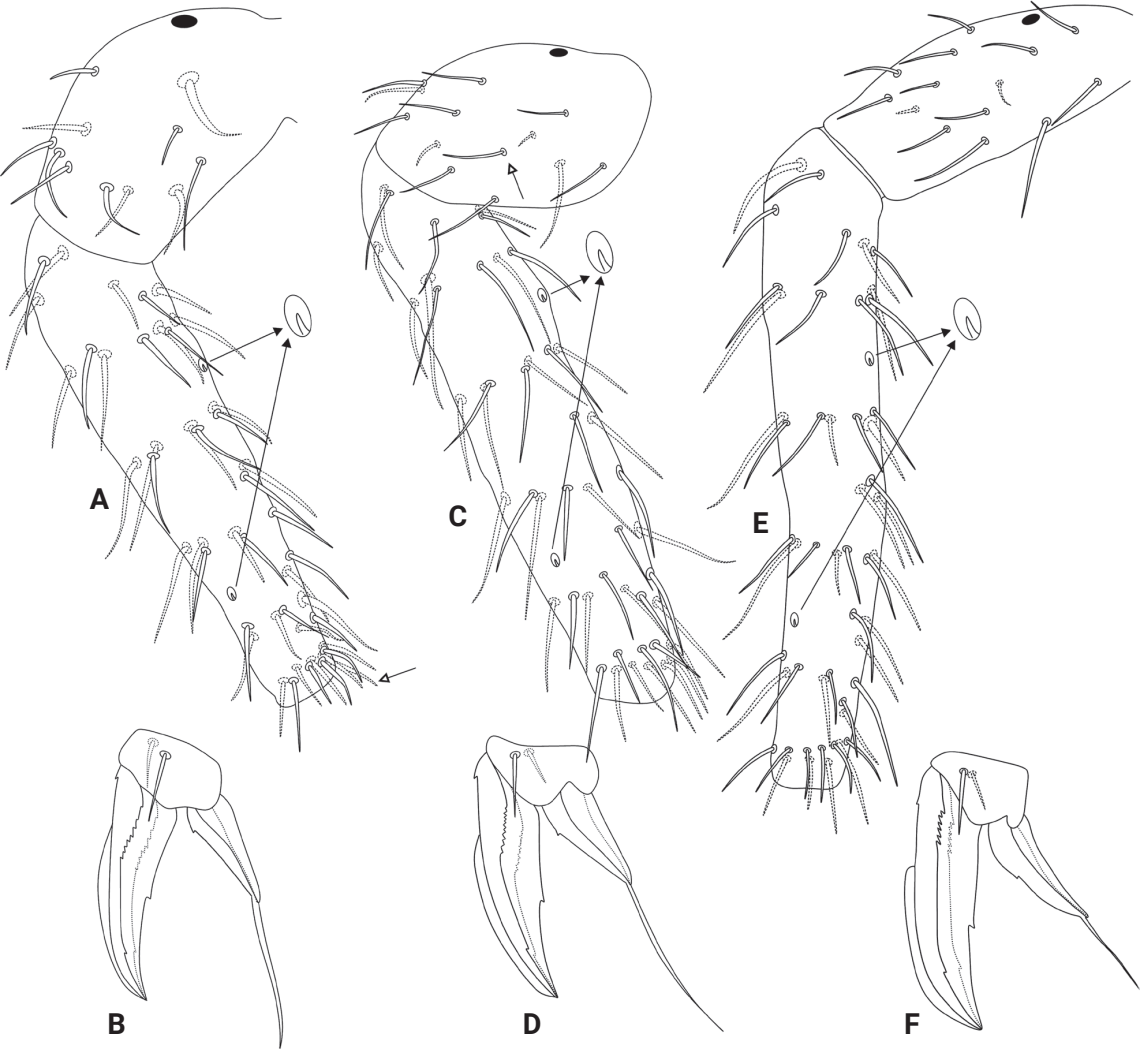
*Szeptyckithecacyanea* sp. nov. legs **A** femur and tibiotarsus I (detail shows oval organs with internal small sensillum) **B** foot complex I **C** femur and tibiotarsus II (detail shows oval organs with internal small sensillum) **D** foot complex II **E** femur and tibiotarsus III (detail shows oval organs with internal small sensillum) **F** foot complex III.

Figs 7 , 8 , 9 , 10 , 11 , Table 1

##### Type material.

***Holotype*** male on slide, Brazil, Rio Grande do Norte state, Nísia Floresta municipality, “Floresta Nacional de Nísia Floresta” (6°5'9.132"S, 35°10'53.857"W), 02/VI/2022, Xavier M.D. col., pitfall traps. ***Paratypes*** on slides: one male and one female, with the same data as the holotype.

##### Other examined material.

Two males and one female on slides, Brazil, Rio Grande do Norte state, Natal municipality, “Mata da CAERN – UFRN” (5°50'9.665"S, 35°12'12.953"W).

##### Diagnosis.

Specimens mostly bluish. Ant. IV with 11 subsegments, with at least six capitate chaetae; Ant. III with 21 chaetae other than the sensory clubs, including two peculiar small sensilla within cavities; Ant. II undivided, with 15 chaetae, four of them slightly longer than the others; Ant. I with six chaetae. Eyepatches with two small interocular chaetae. Head vertex with a total of 18 large spines, two of them unpaired; unpaired chaeta **A1** present and regular (not spine-like); secondarily reduced chaetae near the spines absent. Trochanters I–III with 2,1,1 spines, respectively, trochanters I and II spines capitate, III blunt; trochanter III with five regular chaetae other than the spine. Ungues with a one inner tooth, with tunica and weak pseudonychia; unguiculus I with the internal tooth; unguiculus III filament thin and not reaching the tip of the unguis III. Large abdomen dorsally with ~ 26+26 long capitate mac. Female with a long subanal appendage (surpassing the apex of the ventral anal valves), slightly curved at the apex, acuminate, and apically serrated on its internal face. Manubrium with 7+7 dorsal chaetae; dens ventral chaetotaxy formula from the apex to the base as: 3,2…1, dorsal chaetotaxy with 16 chaetae; mucronal notch prominent.

##### Description.

Body (head + trunk) length of the type series ranging between 551 and 818 µm, holotype with 624 µm, males’ average size = 588 µm, females’ average size = 818 µm, entire type series’ average size = 664.5 µm. Specimens entirely dark bluish (Fig. 7).

Head (Fig. 8). Antennae length 445.4 µm in the holotype. Holotype antennal segment ratio I:II:III:IV as 1:1.3:2.3:5.2. Ant. IV with 11 subsegments, subsegment I with two or three, II with four, III with three or four, IV with five or six, V with eight, VI–X with ten each, and XI with ~ 19 chaetae, respectively, subsegments I+II with six or seven capitate chaetae (Fig. 8A). Ant. III with 21 chaetae other than the sensory clubs, including two peculiar small sensilla within cavities and one small sensillum without cavity, regular chaetae sizes variable but none remarkably longer than the others, most chaetae capitate, sensory rods inside two separate shallow cavities (Fig. 8B). Ant. II undivided, with 15 capitate chaetae, four of them slightly longer than the others (Fig. 8B). Ant. I with six chaetae, one of them ventral (Fig. 8B). Eyes 8+8, with 2+2 small interocular chaetae, head capsule normal (not elongated) (Fig. 8C). Clypeal area **a**–**f** lines with 7–8/7/5/6/5(+1)/3 dorsal + ventral chaetae, respectively, **e1** chaeta present, zones without cuticular granulation and oval organs only seen in the ventral side (Fig. 8C, D). Interantennal area **α** and **γ** lines with 1/2 regular chaetae, respectively; frontal area **A**–**E** lines with a total of 18 large smooth spines, chaetotaxy following the formula: 1(+1)/2/1(+1)/2(+1)/3, respectively, without secondarily reduced spines, **A1** chaeta present (Fig. 8C). Labial basomedian field with four, basolateral field with five chaetae, respectively (Fig. 8D). Maxillary outer lobe with apical chaeta subequal to the basal chaeta, none barbed, sublobal plate with one chaeta-like appendage (Fig. 8E). Labial palp with six proximal chaetae, formula of the guards: **H**(2), **A**(0), **B**(5), **C**(0), **D**(4), **E**(5) plus the lateral process (Fig. 8F). Six prelabral chaetae present (Fig. 8C); labral **p**, **m**, and **a** lines with 5, 5, 4 chaetae, respectively, **p2** longer than the others, labral intrusions present, labral papillae absent, labrum apically without clear modifications (Fig. 8G). Mandibles normal (not elongated), with 5+4 incisive apical teeth (Fig. 8H). Maxilla capitulum elongate (Fig. 8I).

Trunk (Fig. 9). Large abdomen: thorax continuous with the abdomen, without segmentations. Th. II with one **a** small chaeta and three blunt spines on **m** line; Th. III with one capitate **a**, two **m** and one **p** chaetae, **p** reduced in males and elongate in females; Abd. I with one **a**, one **m** and one **p** chaetae; Abd. II bothriotricha **A**, **B**, and **C** slightly misaligned, **C** clearly longer than **A** and **B**, with three **a**, five **m**, and seven **p** chaetae of different shapes near the bothriotricha, at least two of them clearly shorter in males; Abd. II with seven long capitate mac. Abd. III and IV with four main lines of chaetae above the bothriotrichum **C**: **dI-1** with five, **dII-1** with six, **dIII-1** with three and **dIV-1** with one capitate chaetae, respectively, female with some chaetae longer than on male (Fig. 9A). Parafurcal area with four rows of chaetae, with three, three, two or three and four chaetae, respectively, neosminthuroid chaeta present. Three extra capitate chaetae between the bothriotrichum **C** and the parafurcal area (Fig. 9A). Small abdomen: including Abd. V–VI in both sexes. Abd. V chaetae smooth, with bothriotrichum **D** with one small accessory chaeta, chaeta above bothriotrichum **D** elongate in females, absent in males (Fig. 9B, D), Abd. VI chaetae apparently smooth. Female Abd. VI: dorsal anal valve with **as1**–**4**, **ams1**, **ms1**–**4**, **mps1**–**3**, and **ps1**–**2** chaetae, **as1**, **ams1**, **ms1**, and **ps1** unpaired; each ventral anal valve with **ai1**–**6**, **ami1** (as an oval organ), **mi1**–**5**, **mpi1**–**2**, and **pi1**–**3** chaetae, **mi5** as the subanal appendage, long (surpassing the apex of the ventral anal valves), slightly curved at the apex, acuminate, and apically serrated on its internal face (Fig. 9B). Female genital plate with 4+4 ventral chaetae (Fig. 9C). Male Abd. VI: dorsal anal valve with **as2**–**4**, **ms1**–**4**, **mps2**, and **ps1**–**2** chaetae, **ms1** and **ps1** unpaired; each ventral anal valve with **ai1**–**4**, **ami1** (as an oval organ), **mi1**–**5**, **mpi2**, and **pi1**–**3** chaetae (Fig. 9D). Male genital plate with 14+14 chaetae (Fig. 9E).

Abdominal appendages (Fig. 10A–C). Ventral tube with 1+1 chaetae on the lateral flaps, sacs long and warty. Tenaculum ramus with three teeth each plus an apically rounded basal appendix, corpus with 2+2 chaetae. Manubrium with 7+7 dorsal chaetae (Fig. 10A); dens ventrally (anteriorly) with six chaetae, following the formula from the apex to the basis: 3,2...1 (Fig. 10B); dens dorsally without basal appendages, with 16 dorsal (posterior) chaetae (Fig. 10C); Mucro short, apically split, external lamella serrated (with 12–18 serrations), internal with two distal weak crenulations, ending in a prominent apical notch (Fig. 10C). Manubrium:dens:mucro ratio of the holotype = 1.05:2.4:1.

Legs (Figs 10D–F, 11). Leg I: epicoxa with one chaeta, subcoxa and coxa without chaetae; trochanter with two capitate spines plus two capitate chaetae (Fig. 10D); femur with one oval organ, one acuminate large curved spine and ten regular chaetae; tibiotarsus with two oval organs and 46–47 chaetae, nine or ten of them in the apical whorl (Fig. 11A); pretarsus with anterior chaeta longer than the posterior one, unguis with one internal, two lateral and one dorsal teeth, with tunica and weak pseudonychia, unguiculus with the tooth, apical filament thin and surpassing the tip of the unguis (Fig. 11B). Leg II: epicoxa and subcoxa with one chaeta each, coxa with two chaetae, one of them curved, other capitate; trochanter with one thick capitate spine and four regular chaetae (Fig. 10E); femur with one oval organ, two reduced and 10–11 regular chaetae; tibiotarsus with two oval organs and 47 chaetae, ten of them in the apical whorl (Fig. 11C); pretarsus with anterior chaeta longer than the posterior one, unguis with one internal, two lateral and one dorsal teeth, with tunica and weak pseudonychia, unguiculus with the tooth, apical filament thin and surpassing the tip of the unguis (Fig. 11D). Leg III: epicoxa and subcoxa with one chaeta each, coxa with four chaetae; trochanter with one thick blunt spine, one oval organ and five regular chaetae (Fig. 10F); femur with one oval organ, two reduced and 12 regular chaetae; tibiotarsus with two oval organs and 50 chaetae, ten of them in the apical whorl (Fig. 11E); pretarsus with anterior chaeta longer than the posterior one, unguis with one internal, two lateral and one dorsal teeth, with tunica and weak pseudonychia, unguiculus with the tooth, apical filament thin and not reaching the tip of the unguis (Fig. 11F); tibiotarsi oval organs with reduced inner sensilla (Fig. 11A, C, E). Ratio of ungues I–III in the holotype = 1:1.05:1.04.

##### Etymology.

The species was named after its color pattern; *cyanea* from Latin means dark blue.

##### Habitat.

Specimens of *S.cyanea* sp. nov. were found in two localities ~ 30 km apart in the Rio Grande do Norte state, Brazil: in the central campus of the Federal University of Rio Grande do Norte, Natal municipality, and the National Forest of Nísia Floresta, Nísia Floresta municipality (Fig. 6). Both localities are inserted in the Atlantic Forest phytogeographic domain. The specimens were collected from the forest litter covering sandy soils in shady areas. The climate of the sampled municipalities is “As” following the Köppen-Geiger system, which means an equatorial climate with dry summer (Kottek et al. 2006). The specimens were collected during the raining season of 2022.

##### Remarks.

*Szeptyckithecacyanea* sp. nov. is the sole species of the genus with 18 spines on the head vertex. It is also the only Neotropical *Szeptyckitheca* with the frontal head A1 chaeta (see Table 1). Considering the Neotropical fauna, it is somewhat similar to *S.andrzeji* sp. nov. in the number of Ant. IV subsegments (11), Ant. I chaetae (6), and the presence of two capitate trochanteral spines on the leg I. However, they differ in the previously mentioned features, as well as the presence of secondarily reduced chaetae on the head frons of *S.andrzeji* sp. nov. (absent in *S.cyanea* sp. nov.), female subanal appendage morphology (short and spoon-like in *S.andrzeji* sp. nov., long and acuminate in *S.cyanea* sp. nov.) and ventral dens chaetotaxy formula (3…1 in *S.andrzeji* sp. nov., 3,2…1 in *S.cyanea* sp. nov.), among other characteristics. Further comparisons are presented in Tables 1 and 2.

### ﻿Identification key of *Szeptyckitheca* species*

**Table:** 

1	Head vertex with a set of 9 rough curved mac plus 4 large erect spines	***S.koreana* (Betsch & Weiner, 2009), comb. nov. (North Korea)**
–	Head vertex without rough curved mac, with 6 or more large erect spines	**2**
2	Head vertex with 15 large spines, ventral dens chaetotaxy formula as: 3,1…1, ungual pseudonychia absent	***S.formosana* (Yosii, 1965) (Taiwan)**
–	Head vertex with 6–14 or 16–18 large spines, ventral dens chaetotaxy formula otherwise, ungual pseudonychia usually present	**3**
3	Head vertex with 10 large spines, ungual tunica absent or at most vestigial, female subanal appendage very long, surpassing the dorsal anal valve	***S.boneti* (Denis, 1948) (Vietnam)**
–	Head vertex with 6 or 11–18 spines, ungual tunica present, female subanal appendage not reaching the apex of the dorsal anal valve	**4**
4	Head clypeal **f** line with 4+4 mac, ventral dens chaetotaxy formula as: 4,1…1, dorsal dens with 15 chaetae	***S.kac* Zeppelini, Lopes & Lima, 2018 (Brazil)**
–	Head clypeal **f** line without mac, ventral dens chaetotaxy formula otherwise, dorsal dens with 12–14 or 16–24 chaetae	**5**
5	Trochanter II without the spine, dorsal dens with 24 chaetae	***S.machadoi* (Delamare-Deboutteville & Massoud, 1964) (Angola, Congo)**
–	Trochanter II usually with the spine, dorsal dens with ≤ 20 chaetae	**6**
6	Head interantennal area with bifid chaetae, female anal valve with **mps1** winged, dorsal dens with 20 chaetae	***S.kesongensis* Betsch & Weiner, 2009 (North Korea)**
–	Head interantennal area without bifid chaetae, female anal valve with **mps1** not winged, dorsal dens with < 20 chaetae	**7**
7	Head vertex with 6 large spines, ventral dens chaetotaxy formula as: 4,2…1	***S.coerulea* (Bretfeld 2005) (Yemen - Socotra Island)**
–	Head vertex with 11 or more large spines, ventral dens chaetotaxy formula otherwise	**8**
8	Specimens pale, with diffused pigment and violet patches, head vertex with 11 large spines	***S.nepalica* (Yosii, 1966) (Nepal)**
–	Specimens color pattern otherwise, head vertex with 14–18 large spines	**9**
9	Specimens with lateral weak purple bands, posteriorly purple, dorsal dens with 14 chaetae	***S.mucroserrata* (Snider, 1978) (Brazil, Mexico, USA)**
–	Specimens color pattern otherwise, dorsal dens with 12–13 or 16–19 chaetae	**10**
10	Trochanter II without the spine, dorsal dens with 12 chaetae	***S.karlarum* (Palacios-Vargas, Vázquez & Cuéllar, 2003), comb. nov. (Mexico)**
–	Trochanter II with the spine, dorsal dens with > 12 chaetae	**11**
11	Ungues without the inner tooth, dorsal dens with 13 chaetae	***S.santiagoi* (Yosii, 1959) (Australia, Papua New Guinea, Singapore, Solomon Isl)**
–	Ungues with 1 inner tooth, dorsal dens 16–19 chaetae	**12**
12	Head frontal chaeta **A1** present	**13**
–	Head frontal chaetae **A1** absent	**14**
13	Head vertex with 18 large spines, eyepatches with 2 interocular chaetae, ventral dens chaetotaxy formula as: 3,2…1, dorsal dens with 16 chaetae	***S.cyanea* Oliveira, Medeiros & Bellini, sp. nov. (Brazil)**
–	Head vertex with 16 large spines, eyepatches with 1 interocular chaeta, ventral dens chaetotaxy formula as: 2,2…1, dorsal dens with 19 chaetae	***S.implicata* (Hüther, 1967) (South Sudan)**
14	Female subanal appendage short (not reaching the apex of the ventral anal valves), ventral dens chaetotaxy formula as: 3…1	***S.andrzeji* Medeiros, Bellini & Weiner, sp. nov. (Brazil)**
–	Female subanal appendage long (surpassing the apex of the ventral anal valves), ventral dens chaetotaxy formula as: 3–2,2…1	**15**
15	Head vertex with 14 large spines, trochanter I with 2 spines, unguiculus I without the internal tooth, mucronal notch prominent	***S.bellingeri* (Betsch, 1965) (Jamaica)**
–	Head vertex with 16 large spines, trochanter I with 1 spine, unguiculus I with the internal tooth, mucronal notch discrete	**16**
16	Ant. II with 14 chaetae, trochanter III spine capitate, with 4 regular chaetae, ventral dens chaetotaxy formula as: 3,2…1, dorsal dens with 17 chaetae	***S.peteri* (Palacios-Vargas, Vázquez & Cuéllar, 2003), comb. nov. (Mexico)**
–	Ant. II with 12 chaetae, trochanter III spine blunt, with 5 regular chaetae, ventral dens chaetotaxy formula as: 2,2…1, dorsal dens with 16 chaetae	***S.vanderdrifti* (Delamare-Deboutteville & Massoud, 1964) (Suriname)**

*We did not include *S.spinimucronata* as we considered it a *species inquirenda*. Further details on the species are listed in its diagnosis.

## ﻿Discussion

The boundaries between *Szeptyckitheca* and *Sphyrotheca* are quite narrow (Betsch and Weiner 2009). Many chaetotaxic features are variable within *Szeptyckitheca*, like the presence or absence of frontal chaeta **A1** on head, ungual tunica and even the ventral dental chaetae formula (see Tables 1, 2), and so are not unequivocal to separate these genera. The same occurs in *Sphyrotheca*, and for instance, the presence of apically curved mac (spines) on head’s frontal area and dorsal large abdomen listed in Betsch and Weiner (2009) differs within the genus, being present in species like *Sphyrothecamultifasciata* (Reuter, 1881), and absent in Neotropical taxa like *Sphyrothecacaputalba*Bretfeld 2002 (Bretfeld 2000, 2002). With the description of *Szeptyckithecaandrzeji* sp. nov., with its *Sphyrotheca*-like reduced ventral dens chaetotaxy, and the transfer of *Sphyrothecakoreana* to *Szeptyckitheca* due to the presence of trochanteral spines on all legs, the ventral dens chaetotaxy and the presence of curved mac on frontal head and dorsal large abdomen do not allow to separate the two genera, and the sole morphological trait which clearly differs between them is the presence of the trochanteral spine on leg 1 of *Szeptyckitheca* species. This observation supports a close relationship between these genera and it is possible one of them emerged from the other. A molecular phylogenetic analysis of the Sphyrothecinae is in need to corroborate or reject this hypothesis and to reinforce (or dismiss) the validity of *Szeptyckitheca*.

After the comparison of both new species described here, we observed other features which may be useful to diagnose *Szeptyckitheca* taxa, such as the shape of the maxilla capitulum (globose vs elongate), the shape of **E** chaetae on frontal head, the presence of an extra basal appendix on proximal dens and the number, and homology of clypeal unpaired chaetae. These features were not used in this study to separate the species due to the lack of information or uncertainties about such data in most (or all) previously described species. Nevertheless, with the expansion of the knowledge of the genus they may be useful to identify closely related taxa.

After our study, there are now 18 species assigned to *Szeptyckitheca*. Even so, many of them are in need of revision or full redescription.

## Supplementary Material

XML Treatment for Szeptyckitheca

XML Treatment for Szeptyckitheca bellingeri

XML Treatment for Szeptyckitheca boneti

XML Treatment for Szeptyckitheca coerulea

XML Treatment for Szeptyckitheca formosana

XML Treatment for Szeptyckitheca implicata

XML Treatment for Szeptyckitheca kac

XML Treatment for Szeptyckitheca karlarum

XML Treatment for Szeptyckitheca kesongensis

XML Treatment for Szeptyckitheca koreana

XML Treatment for Szeptyckitheca machadoi

XML Treatment for Szeptyckitheca mucroserrata

XML Treatment for Szeptyckitheca nepalica

XML Treatment for Szeptyckitheca peteri

XML Treatment for Szeptyckitheca santiagoi

XML Treatment for Szeptyckitheca spinimucronata

XML Treatment for Szeptyckitheca vanderdrifti

XML Treatment for Szeptyckitheca andrzeji

XML Treatment for Szeptyckitheca cyanea

## ﻿Additional information

### Author ORCIDs

Bruno Cavalcante Bellini https://orcid.org/0000-0001-7881-9436

Wanda Maria Weiner https://orcid.org/0000-0002-7257-3671

Rudy Camilo Nunes https://orcid.org/0000-0002-3140-9146

Gleyce Da Silva Medeiros https://orcid.org/0000-0001-9839-2345

### Data availability

All of the data that support the findings of this study are available in the main text.
